# Mutagenesis-Based Characterization and Improvement of a Novel Inclusion Body Tag

**DOI:** 10.3389/fbioe.2019.00442

**Published:** 2020-01-10

**Authors:** Wouter S. P. Jong, Corinne M. ten Hagen-Jongman, David Vikström, Wendy Dontje, Abdallah M. Abdallah, Jan-Willem de Gier, Wilbert Bitter, Joen Luirink

**Affiliations:** ^1^Abera Bioscience AB, Solna, Sweden; ^2^Department of Molecular Microbiology, Amsterdam Institute for Molecules Medicines and Systems (AIMMS), Vrije Universiteit, Amsterdam, Netherlands; ^3^Xbrane Biopharma AB, Solna, Sweden; ^4^Department of Clinical Immunology and Rheumatology, Amsterdam UMC, University of Amsterdam, Amsterdam, Netherlands; ^5^Department of Basic Medical Sciences, College of Medicine, QU Health, Qatar University, Doha, Qatar; ^6^Bioscience Core Laboratory, King Abdullah University of Science and Technology (KAUST), Jeddah, Saudi Arabia; ^7^Department of Biochemistry and Biophysics, Center for Biomembrane Research, Stockholm University, Stockholm, Sweden; ^8^Medical Microbiology and Infection Control, Cancer Center Amsterdam, Amsterdam UMC, Vrije Universiteit, Amsterdam, Netherlands

**Keywords:** inclusion bodies, fusion tag, insoluble expression, protein aggregation, heterologous protein production, signal peptide, twin-arginine translocation pathway, chaperone

## Abstract

Whereas, bacterial inclusion bodies (IBs) for long were regarded as undesirable aggregates emerging during recombinant protein production, they currently receive attention as promising nanoparticulate biomaterials with diverse applications in biotechnology and biomedicine. We previously identified ssTorA, a signal sequence that normally directs protein export via the Tat pathway in *E. coli*, as a tag that induces the accumulation of fused proteins into IBs under overexpression conditions. Here, we used targeted mutagenesis to identify features and motifs being either critical or dispensable for IB formation. We found that IB formation is neither related to the function of ssTorA as a Tat-signal sequence nor is it a general feature of this family of signal sequences. IB formation was inhibited by co-overexpression of ssTorA binding chaperones TorD and DnaK and by amino acid substitutions that affect the propensity of ssTorA to form an α-helix. Systematic deletion experiments identified a minimal region of ssTorA required for IB formation in the center of the signal sequence. Unbiased genetic screening of a library of randomly mutagenized ssTorA sequences for reduced aggregation properties allowed us to pinpoint residues that are critical to sustain insoluble expression. Together, the data point to possible mechanisms for the aggregation of ssTorA fusions. Additionally, they led to the design of a tag with superior IB-formation properties compared to the original ssTorA sequence.

## Introduction

Heterologous proteins that are overexpressed in microbial hosts such as *E. coli* often accumulate in insoluble clusters called inclusion bodies (IBs). The IBs preferentially localize at the poles of the bacteria, which is thought to be related to the accumulation of dense DNA central in the cells. The IBs vary in size from 0.1 to 1 μm and although their chemical composition may vary, they consist predominantly of the overexpressed recombinant protein (Ramon et al., 2014; de Marco et al., 2019). Traditionally, IB formation is linked to the aggregation of misfolded polypeptides and seen as an obstacle in production processes aimed at high yields of soluble, active protein species. To avoid aggregation several strategies have been explored including lowering of transcription and translation rates, reduction of the growth temperature and co-expression of chaperones that assist in proper folding (Kaur et al., 2018). Alternatively, IBs can be isolated, denatured and subjected to refolding conditions to regenerate the active protein (Yamaguchi and Miyazaki, 2014; Singh et al., 2015).

Rather than being regarded as an undesirable side effect of industrial protein production, the generation of IBs is now increasingly appreciated as a useful strategy to obtain recombinant protein. Proteins in IBs are largely resistant against degradation by host cell proteases and less likely to exert toxic effects in the expression host. In addition, owing to their high density, IBs are easy to isolate from cell lysates by differential centrifugation, providing robust and cost-efficient protocols to obtain large amounts of relatively pure protein (Swartz, 2001; Sahdev et al., 2008; Martinez-Alonso et al., 2009). Moreover, whereas IBs for long were considered to be amorphous aggregates of disordered protein, they actually display a structured β-sheet organization and can contain significant amounts of protein in a native conformation. This makes them interesting as a supply of nanostructured biomaterial with high mechanical stability, biological activity and slow protein release properties. These IBs may for example be exploited as immobilized reusable catalysts in biotechnology or as “nanopills” for the delivery of therapeutics and vaccines in biomedicine (Ramon et al., 2014; de Marco et al., 2019).

Unfortunately, the formation of IBs, approaches to prevent their formation and conditions for refolding proteins from IBs are difficult to predict and need to be evaluated and addressed on a case-to-case basis. Different parameters may impact aggregation such as the extent and kinetics of induced expression, culture conditions (temperature, growth rate, pH) and the presence or absence of folding modulators in the host strain. The same parameters may also affect the size of the inclusion bodies, their physical properties and the balance between denatured and native protein (Rinas et al., 2017; Slouka et al., 2019).

To increase the yield of inclusion bodies and allow a more robust and predictable workflow, fusion tags have been developed that induce aggregation of the recombinant protein. A well-known example is the strongly hydrophobic ketosteroid isomerase sequence (Kuliopulos and Walsh, 1994) that is commercially available in the pET31b expression vector. Other examples include trpΔLE (Derynck et al., 1984), β-galactosidase (Schellenberger et al., 1993), PagP (Hwang et al., 2012), EDDIE (Achmuller et al., 2007), ELK16 (Wu et al., 2011), GFIL8 (Wang et al., 2015), PaP3.30 (Rao et al., 2004), TAF12-HFD (Vidovic et al., 2009), and the F4 fragment of PurF (Lee et al., 2000). Most described tags comprise relatively large sequences (Derynck et al., 1984; Schellenberger et al., 1993; Kuliopulos and Walsh, 1994; Lee et al., 2000; Rao et al., 2004; Achmuller et al., 2007; Hwang et al., 2012) that may adversely affect the yield of the target protein or compromise downstream applications. Smaller IB-tags have been presented but were only used for the production of short polypeptides (Vidovic et al., 2009) or the functionality of the tag was difficult to evaluate (Cook et al., 2011; Wu et al., 2011; Zhou et al., 2012; Wang et al., 2015).

Previously, we have serendipitously found that the 39-amino acid signal sequence of *E. coli* TMAO-reductase (ssTorA) functions as a small and robust, versatile fusion tag that promoted IB formation of any polypeptide it was fused to, ranging from small human hormones to very large bacterial secretory proteins. Interestingly, fusion to GFP yielded fluorescent aggregates, alluding to a compatibility of ssTorA with the production of bioactive IBs. Evidence was further obtained that multiple ssTorAs fused in tandem either at the N-terminus, C-terminus or both, can mediate complete aggregation of even very soluble cargo proteins such as maltose binding protein (Jong et al., 2017).

In this study we investigated the minimal requirements and mechanism by which ssTorA, a peptide that is not particularly hydrophobic and normally triggers transport of folded proteins across the bacterial inner membrane via the twin-arginine translocation (Tat) pathway (Sargent, 2007), achieves robust induction of inclusion body formation. We identified a region central in ssTorA that is critical for IB formation. In fact, a shortened IB-tag based on this information provoked increased expression and IB formation compared to the original ssTorA. Further studies pointed to the importance of specific residues in ssTorA and the role of specific and generic chaperones in ssTorA induced IB formation.

## Materials and Methods

### Strains, Media, and Growth Conditions

*Escherichia coli* K-12 strain TOP10F′ (Invitrogen, UK) and MC4100 (Casadaban, 1976) were used for plasmid-based protein expression. MC4100Δ*tatA/E* (Sargent et al., 1998) was used when indicated. When grown in culture, cells were grown in LB medium in a shake incubator in the presence of ampicillin (100 μg/ml). Cultures of cells containing pOFX-*tac* (derivative) plasmids also contained kanamycin (50 μg/ml) and glucose (0.2%). Cultures were grown at 37°C in tubes or flasks in a shake incubator (200 rpm) using a 5:1 tube/flask:culture volume ratio. Growth on solid medium was performed at 37°C making use of LB-agar (1.5%) plates supplemented with ampicillin (100 μg/ml). Plates also contained chloramphenicol (30 μg/ml) if indicated.

### Reagents

The Rapid Dephos & Ligation Kit was obtained from Roche Applied Science. Phusion High Fidelity DNA polymerase was purchased from New England Biolabs (New England Biolabs; NEB). Low fidelity DNA polymerase Pfu-Pol(exo^−^) D473G (Biles and Connolly, 2004) was obtained as a kind gift from B. Connolly (University of Newcastle, Newcastle upon Tyne, UK). DNA restriction enzymes were purchased from Roche or NEB. Anhydrotetracyclin was from IBA GmbH. Isopropyl β-D-1-thiogalactopyranoside (IPTG) as purchased from Bioline Reagents Ltd. Lysozyme and all other chemicals were purchased from Sigma Aldrich.

### Plasmid Construction

The plasmids and primers used in this study can be found in Supplementary Tables 2, 3, respectively. Details on plasmid construction can be found as Supplementary Materials and Methods.

### General Protein Expression and Analysis

Plasmid-based protein expression was induced using anhydrotetracycline (0.2 μg/ml) when cell cultures reached an OD_660_ of ~0.3. For analysis, cells and cell-fractions were resuspended in SDS-sample buffer (125 mM Tris–HCl pH 6.8, 4% SDS, 20% glycerol, 0.02% bromophenolblue, 83 mM DTT) and incubated at 96°C for 10 min. Proteins were analyzed by SDS-PAGE and Coomassie Brilliant Blue G (Jansen Chimica) staining. Commercial Bis–Tris NuPAGE (Invitrogen) or TGX gels (Biorad) were used where appropriate. Imaging of Coomassie-stained gels was carried out using a GS-800 densitometer (Biorad) in combination with Quantity-One software (Biorad).

### IB Sedimentation Assay

To separate IBs from the soluble cell content, a culture volume containing the number of cells that gives an OD_660_ of 1.5 in a 1 ml suspension was subjected to centrifugation. The pelleted intact cells were resuspended in 750 μl ice-cold lysis buffer (5 mM Tris–HCl, pH 7.6, 1 mM EDTA, 100 mM NaCl). Lysozyme was added to a final concentration of 17 ng/ml and cells were incubated on ice for 15 min. Subsequently, the cells were disrupted by freeze thawing and tip sonication (Branson Sonifier 250). The resulting lysate was centrifuged (4,500 × g, at 4°C for 10 min) to sediment IBs and other dense, insoluble material. The resulting pellet was subjected to SDS-PAGE analysis directly, whereas the supernatant was trichloroacetic acid precipitated first. Intact cells directly subjected to SDS-PAGE analysis served as a control for total cell content.

### Phase-Contrast Microscopy

For microscopy analysis, cells were fixed in-culture with formaldehyde (3%) at room temperature for 15 min, collected by low-speed centrifugation and re-suspended in PBS. Cells were then photographed with an Olympus F-view II camera mounted on an Olympus 5 BH-2 microscope through a DApo100UV PL 1.30 oil 160/0.17 objective.

### qPCR

Cells carrying either pIBA-ssTorA/TrxA, pIBA-ssTorA(Δ5–9)/TrxA, or pIBA-ssTorA(Δ5–34)/TrxA were induced with anhydrotetracycline for 1 h. Total RNA was isolated from 1.5 OD_660_ units of cells using the RNeasy Mini kit (Qiagen), RNAprotect Bacteria reagent (Qiagen) and DNaseI treatment (Qiagen) according to manufacturer's protocols. For quantitative PCR (qPCR), we used an iCycler and SYBR green Supermix (Bio-Rad, Hercules, CA, US). Expression of *trxA* in each sample was analyzed in duplicates, and expression levels were normalized to the expression of *bla* gene present as an antibiotic resistance marker on the pIBA vector backbone. Primer pairs used were qPCR TrxA fw/qPCR TrxA rv and qPCR bla fw/qPCR bla rv both yielding ~160 bp products.

### ssTorA Random Mutagenesis Library

To create a library of randomly mutagenized DNA encoding ssTorA, error-prone PCR was carried out using Pfu(exo^−^) D473G polymerase, Pfu ultra HF reaction buffer (Agilent), 600 μM of dNTPs and 20 mM of added MgSO_4_. Plasmid pIBA-ssTorA/TrxA (Jong et al., 2017) was used as the template and the primers were ssTorA mut fw and ssTorA mut rv. The PCR product was *Xba*I/*Nhe*I digested and the resulting ssTorA^mut^ fragment–encoding residues 1–36 of ssTorA and the upstream Shine-Dalgarno sequence of the template—was ligated to the corresponding sites of pIBA-ssTorA/CAT. Following electroporation of the ligation mixture into *E. coli* TOP10F′ cells, bacterial colonies were scrape-harvested from LB-agar plates containing ampicillin and pooled. The resulting cell material was suspended in LB with glycerol (12%) to establish a TOP10F′(pIBA-ssTorA^mut^/CAT) library. Prior to harvesting, plasmid DNA from individual colonies was subjected to DNA sequencing and a mutation rate of 1–5 mutations per ssTorA encoding fragment was established for 90% of the pIBA-ssTorA^mut^/CAT ligation products.

### Chloramphenicol Survival Screening and MiSeq Sequencing

Liquid medium was inoculated with part of the TOP10F′(pIBA-ssTorA^mut^/CAT) library cell suspension to an OD_660_ of 0.05 and incubated at 37°C with shaking. When the culture reached an OD_660_ value of 0.3 cells were induced for expression of ssTorA^mut^/CAT by addition of anhydrotetracycline (0.2 μg/ml) and growth was continued. After 2 h samples were withdrawn from the culture and spread on solid medium with or without chloramphenicol (30 μg/ml), respectively, and the resulting plates were incubated at 37°C. To allow comparison of mutational loads in cells grown in the presence or absence of selective pressure, similar numbers of colonies (10,000 and 16,000 CFUs) were harvested from plates with and without chloramphenicol. For either condition, colonies that formed overnight were scrape-harvested and pooled. Plasmid DNA was isolated (GeneJet, ThermoFisher Scientific) from 5 OD_660_ units of cells of either suspension as well as from 5 OD_660_ units of original TOP10F′ (pIBA-ssTorA^mut^/CAT) library cells not subjected to the chloramphenicol survival assay. Using the respective plasmid isolates as a template, a 222 bp fragment including the ssTorA region of interest was PCR amplified using primers ssTorA MiSeq fw and ssTorA MiSeq rv. Following QIAquick gelextraction (Qiagen, Hilden, Germany) the amplicons were purified using the Agencourt AMPure XP PCR Purification system (Beckman Coulter, Pasadena, CA, US) and the resulting starting DNA library was quantified using the Qubit dsDNA BR Assay system (Invitrogen, Carlsbad, CA, US). The sequencing library construction was performed according to the TruSeq Nano DNA sample prep kit (Illumina, San Diego, CA, US). The barcoded libraries were subsequently mixed in equimolar amounts. The multiplexed library pool was spiked with 40% PhiX control to improve base calling during sequencing and was loaded at 7.5 pM. Sequencing was conducted using a paired-end, 2 × 250-bp cycle run on an Illumina MiSeq sequencing system and MiSeq Reagent Kit V2 (500 Cycle) chemistry. After sequencing was complete, image analysis, base calling, and error estimation were performed using Illumina Real-Time Analysis software (version 2.2.0.2).

## Results

### The Relationship Between the Tat Pathway and IB Formation

**Figure 1:**
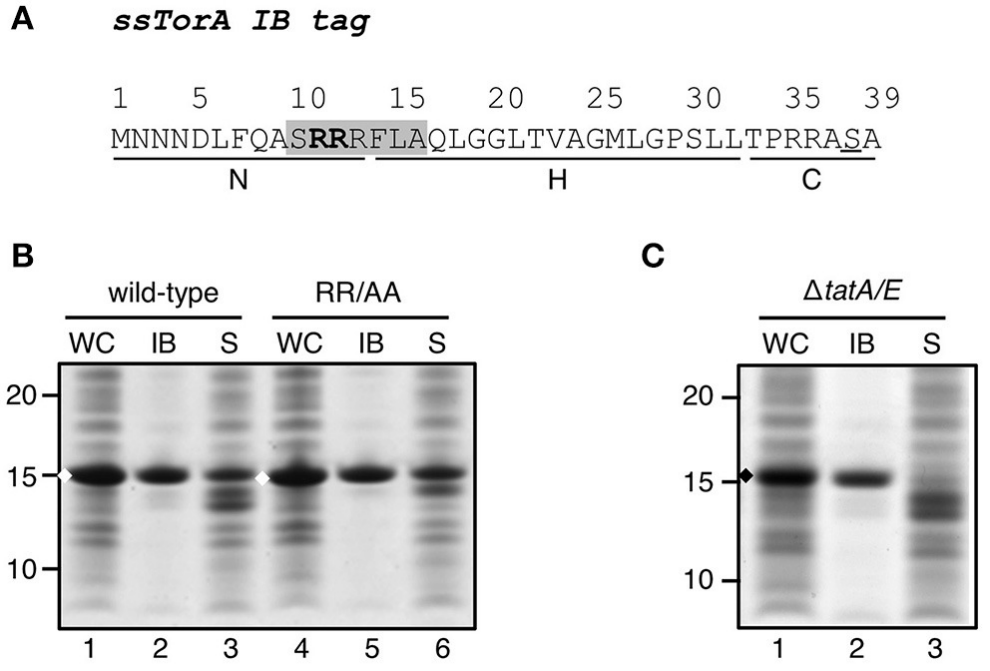
Influence of a functional Tat-system on IB-formation by ssTorA. **(A)**. Amino acid sequence of the ssTorA IB formation tag. The consensus sequence containing a twin-arginine pair (*bold*) that is conserved among Tat-signal sequences is shaded (Berks, 1996). The basic N-region, hydrophobic H-region and polar C-region, which are common for signal sequences (von Heijne, 1985), are indicated below the amino acid sequence (Cristobal et al., 1999). A serine residue that replaces a native threonine in ssTorA (Jong et al., 2017) is underlined. **(B)** IB sedimentation assay on *E. coli* TOP10F′ cells expressing a TrxA fusion carrying wild-type ssTorA as displayed under *A* or derivative ssTorA(RR/AA). Cells were lysed and subjected to differential centrifugation to separate the inclusion bodies (*IB*)-containing insoluble fraction from the soluble cell fraction (*S*). Samples of both fractions and corresponding whole cell (*WC*) samples were analyzed by SDS-PAGE and Coomassie staining. All samples were derived from the same amount of cell material. **(C)** IB-sedimentation assay on MC4100Δ*tatA/E* cells expressing ssTorA/TrxA as described under *B*. Bands representing ssTorA/TrxA fusions of interest are indicated (♦). Molecular mass (kDa) markers are indicated at the left side of the panels.

We previously found the Tat signal sequence of TorA (ssTorA) to function as an inclusion body (IB) tag (Figure 1A) when fused to a cargo protein and expressed at high levels (Jong et al., 2017). To determine whether this capacity is related to its function as a Tat signal sequence, we substituted the twin arginine motif (RR) of ssTorA that is essential for export via the Tat translocon by a non-conservative alanine pair (RR/AA) (Supplementary Table 1). The resulting ssTorA mutant was fused to TrxA that is soluble in *E. coli* even at high concentrations (LaVallie et al., 1993; Jong et al., 2017). IB formation of the fusion proteins was analyzed using an IB sedimentation assay. Briefly, *E. coli* cells expressing the fusion constructs were broken and the lysate was subjected to low-speed centrifugation to separate the dense cell material including IBs from the soluble proteins. The resulting fractions and corresponding whole-cell lysate samples were analyzed by SDS-PAGE and Coomassie staining (Figure 1B). As we demonstrated previously (Jong et al., 2017), the native ssTorA sequence drives efficient IB formation, as evident from the presence of the majority of the ssTorA/TrxA fusion in the low-speed pellet (Figure 1B, lane 2). The RR/AA substitution did not affect the amount of fusion protein in the low speed pellet (Figure 1B, c.f. lane 5 and 2), suggesting that Tat-mediated targeting is not a prerequisite for the functioning of ssTorA as an IB formation tag. Moreover, we expressed ssTorA/TrxA in a strain that lacks the key Tat translocon components TatA and TatE (Sargent et al., 1998). Also in this genetic background, most ssTorA/TrxA fusion protein ended up in the low speed pellet (Figure 1C, lane 2). Together, the data show that ssTorA-mediated IB formation does not require a functional Tat pathway.

**Figure 2:**
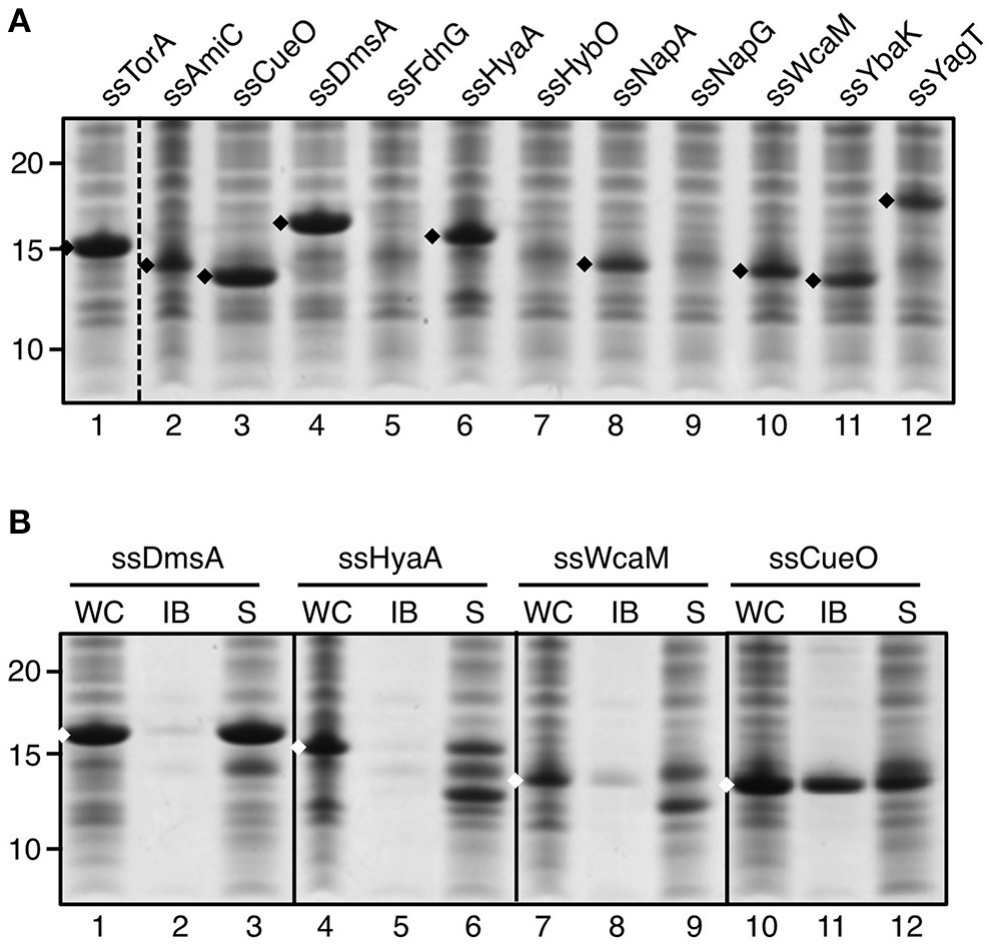
IB formation upon fusion to a panel of Tat signal sequences. **(A)** SDS-PAGE/Coomassie staining analysis on cells overexpressing TrxA fused to a panel of different Tat-signal sequences as indicated. **(B)** IB-sedimentation assay as described in the legend to Figure 1 on cells expressing a selection of the signal sequence/TrxA fusions from *A*. Bands representing signal sequence/TrxA fusions of interest are indicated (♦). Molecular mass (kDa) markers are indicated at the left side of the panels.

To analyze whether the capacity to induce IB formation is a universal feature of Tat signal sequences, a panel of these was selected for fusion to TrxA. The panel represents a cross-section of *E. coli* Tat signal sequences, ranging from relatively short sequences (e.g., ssCueO, 28 aa) to long ones (ssYagT, 53 aa), and including sequences known to bind a cognate chaperone (ssTorA, ssDmsA, FdnG, ssHyaA, ssHybO, NapA) (Chan et al., 2009) and ones that do not (Supplementary Table 1). Despite being cloned in the same expression context, the various signal sequences affected expression differently (Figure 2A). Three chimeras did not show detectable levels of expression (ssFdnG, ssHybO, ssNapG). Another six showed reduced levels compared to the ssTorA/TrxA benchmark (AmiC, ssHyaA, ssNapA, ssWcaM, ssYbaK, ssYagT) whereas two yielded high levels of expression, comparable to the ssTorA/TrxA (ssCueO, ssDmsA). The origin of these differences is unclear, but seems unrelated to length or chaperone binding. Next, we selected TrxA fusions of two chaperone binding (ssDmsA and ssHyaA) and two non-chaperone binding signal sequences (ssCueO and ssWcaM) for analysis of IB formation (Figure 2B). TrxA fused to ssDmsA, ssHyaA, and ssWcaM was predominantly found in the soluble fraction in contrast to ssCueO/TrxA that appeared in the insoluble IB fraction similar to ssTorA/TrxA (Figure 2B, lane 11). The IB induction by ssCueO is surprising as it shares no obvious features with ssTorA. In comparison, ssDmsA seems more closely related to ssTorA with respect to length, hydrophobicity and chaperone binding (Tullman-Ercek et al., 2007; Shanmugham et al., 2012). In conclusion, the features that determine the IB formation capacity of ssTorA are not inherent to all Tat signal sequences.

### Involvement of Cytosolic Chaperones

**Figure 3:**
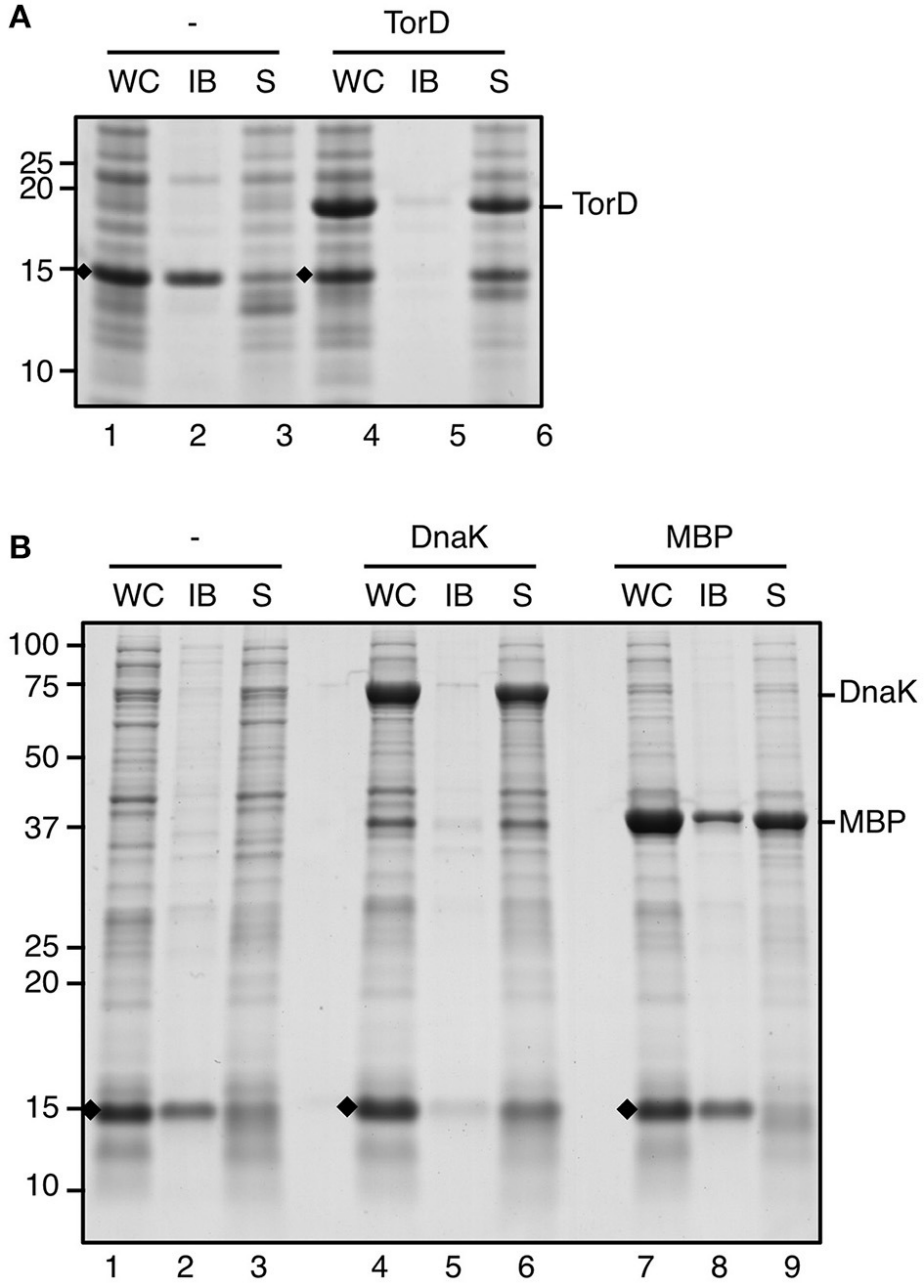
IB formation upon overexpression of chaperones. **(A)** IB sedimentation assay on cells co-transformed with pIBA-ssTorA/TrxA and pLysTac-TorD as described in the legend to Figure 1. Thirty minutes prior to induction of expression of ssTorA/TrxA IPTG was added to half of the culture to induce overexpression of TorD (lanes 4–6). The other half of the culture was left without IPTG (lanes 1–3). **(B)** IB sedimentation assay on cells co-transformed with pIBA-ssTorA/TrxA and either pOFX-tac1-DnaK/DnaJ (lanes 4–6) or pOFX-tac-MBP (lanes 7–9). Thirty minutes prior to induction of expression of ssTorA/TrxA IPTG was added to the respective cultures to induce overexpression of DnaK/DnaJ (lanes 4–6) or MBP (lanes 7–9). Cells co-transformed with pIBA-ssTorA/TrxA and the empty vector pOFX-tac1 served as a negative control for co-overexpression (lanes 1–3). Bands representing ssTorA/TrxA fusions of interest are indicated (♦). Molecular mass (kDa) markers are indicated at the left side of the panels.

In native *E. coli* cells ssTorA functions as a signal sequence to trigger targeting and translocation of TMAO reductase (TorA) via the Tat export pathway (Sargent, 2007). A dedicated cytoplasmic chaperone TorD is co-expressed with TorA when TMAO is present in the medium (Mejean et al., 1994). TorD interacts with ssTorA and co-ordinates the export of the reductase by preventing its premature targeting to the bacterial inner membrane (Hatzixanthis et al., 2005). In our assays, constructs are expressed under conditions of low availability of TorD as no TMAO is added to the growth medium. We therefore considered the possibility that the lack of TorD plays a role in the aggregation of fusions carrying ssTorA. To investigate this, ssTorA/TrxA was produced in cells already expressing high-levels of TorD from a compatible expression plasmid (Figure 3A). Under these co-expression conditions the fusion protein was still efficiently produced but recovered almost exclusively from the soluble cell fraction (Figure 3A, lane 6). In contrast, control cells not induced for TorD expression produced similar amounts of ssTorA/TrxA but located in the insoluble fraction as before (Figure 3A, lane 2). Apparently, overexpressed TorD binds to ssTorA and precludes ssTorA/TrxA IB formation.

Previously, it was shown that ssTorA also interacts with the generic cytosolic chaperone DnaK (Oresnik et al., 2001). Accordingly, using the LIMBO algorithm (Van Durme et al., 2009) DnaK binding sites were predicted for ssTorA at residues 12–18 (RRFLAQL) and 30–36 (SLLTPRR). To investigate whether a disbalance between DnaK and ssTorA under the overexpression conditions used may influence the aggregation process, we monitored IB-formation upon co-overexpression of the chaperone. To this end, ssTorA/TrxA was produced in cells that also express high levels of plasmid-encoded DnaK and its co-chaperone DnaJ (Figure 3B). Insoluble production of ssTorA/TrxA could be confirmed in control cells carrying either an empty co-expression plasmid (Figure 3B, lane 2) or in cells co-overexpressing the unrelated maltose binding protein (MBP) (Figure 3B, lane 8). In contrast, a strongly reduced amount of insoluble ssTorA/TrxA was recovered from cells expressing excess amounts of DnaK/J (Figure 3B, lane 5), indicating impaired IB formation under these conditions. Conceivably, like TorD, DnaK associates with ssTorA and interferes with the aggregation process.

### Regions Critical for IB Formation

**Figure 4:**
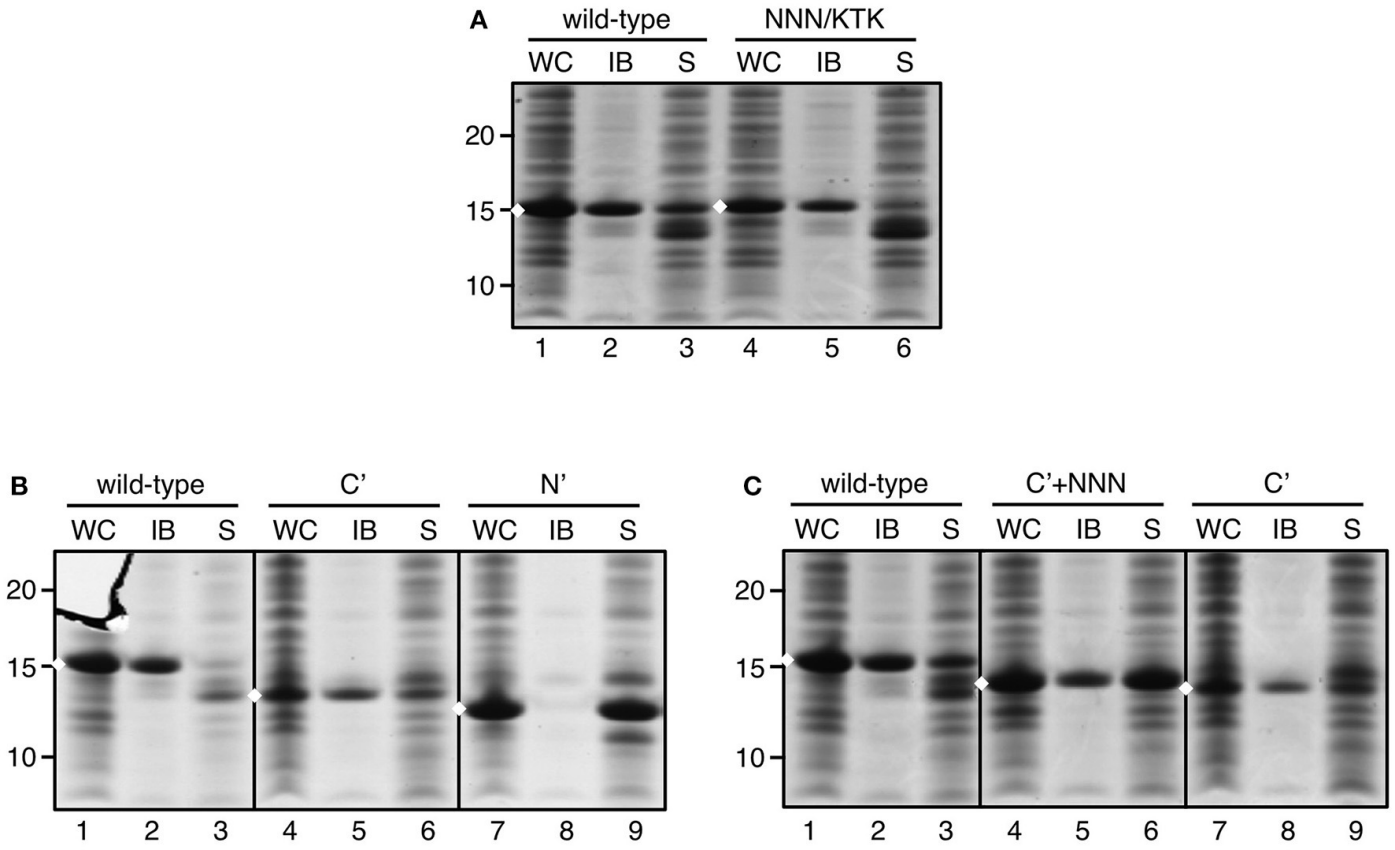
IB formation upon substitution of the NNN motif in ssTorA. IB sedimentation assay as described in the legend to Figure 1 on cells expressing: **(A)** ssTorA/TrxA (*wild-type*) or ssTorA(NNN/KTK)/TrxA, **(B)** ssTorA/TrxA (*wild-type*), ssTorA(C′)/TrxA, or ssTorA(N′)/TrxA, and **(C)** ssTorA/TrxA (*wild-type*), ssTorA(C′+NNN)/TrxA or ssTorA(C′)/TrxA. Bands representing ssTorA/TrxA fusions of interest are indicated (♦). Molecular mass (kDa) markers are indicated at the left side of the panels.

We next focused on sequence motifs and regions not related to the Tat system that influence IB formation by ssTorA. The triple asparagine sequence (NNN) directly downstream of the initiator methionine (Supplementary Table 1) was considered potentially important because prions typically possess a region rich in asparagine and/or glutamine residues that enhances their aggregation (Du, 2011). Initially we substituted the asparagine residues by alanine residues. However, this modification was found to impair expression (data not shown), possibly due to the codon usage in the encoding mRNA. Whereas, in prokaryotes A/T-rich codons are preferred around the start of the coding sequence, alanine codons intrinsically have a high G/C-content, which may interfere with efficient translation initiation and expression (Kozak, 2005). To prevent impaired expression, the NNN motif was substituted with a KTK sequence as present in ssDmsA (Figure 4A; Supplementary Table 1) that sustained high expression levels upon fusion to TrxA but without inducing IB formation (Figure 2). With the NNN/KTK substitution incorporated, expression and IB formation of ssTorA/TrxA was on par with the wild-type ssTorA/TrxA (Figure 4A). *Vice versa*, replacement of the KTK motif of ssDmsA by NNN did not induce insoluble expression of ssDmsA/TrxA fusion (data not shown). Together, the data indicate that the NNN sequence in ssTorA is neither required nor sufficient for IB formation. The presence of A/T rich codons at the start of ssTorA seems important to achieve high-level expression, which likely is a prerequisite for efficient IB formation.

Rather than mutating individual motifs in ssTorA, we next applied an unbiased deletion strategy to identify sequences that are critical for IB formation. We set out making deletions based on the natural N-H-C domain structure of the signal sequence (see Figure 1A). The basic N-domain of ssTorA (AA 2–13) was removed, leaving the C-terminal domain with hydrophobic core and polar C-domain intact [ssTorA(C′)] (Supplementary Table 1). Alternatively, we kept the N-domain intact and removed the C-terminal part of ssTorA (except C-terminal ASA; AA 14–36)[ssTorA(N′)]. A TrxA fusion carrying ssTorA(N′) was exclusively expressed in the soluble fraction (Figure 4B, lane 9). In contrast, ssTorA(C′)/TrxA accumulated in IBs (Figure 4B, lane 5), although the yield appeared relatively low for this construct due to reduced expression (Figure 4B, c.f. lanes 4 and 1). In an attempt to enhance the expression of ssTorA(C′)/TrxA we reinserted the N-terminal NNN motif (Supplementary Table 1), the codon usage of which appeared favorable for translation initiation (see above). Indeed, improved expression of the resulting ssTorA(C′+NNN)/TrxA chimera was observed restoring the IB yield to the level of ssTorA/TrxA (Figure 4C, c.f. lanes 4–5 and 1–2). In conclusion, the C-terminal part of ssTorA (AA 14–39) appears sufficient for induction of IB formation.

**Figure 5:**
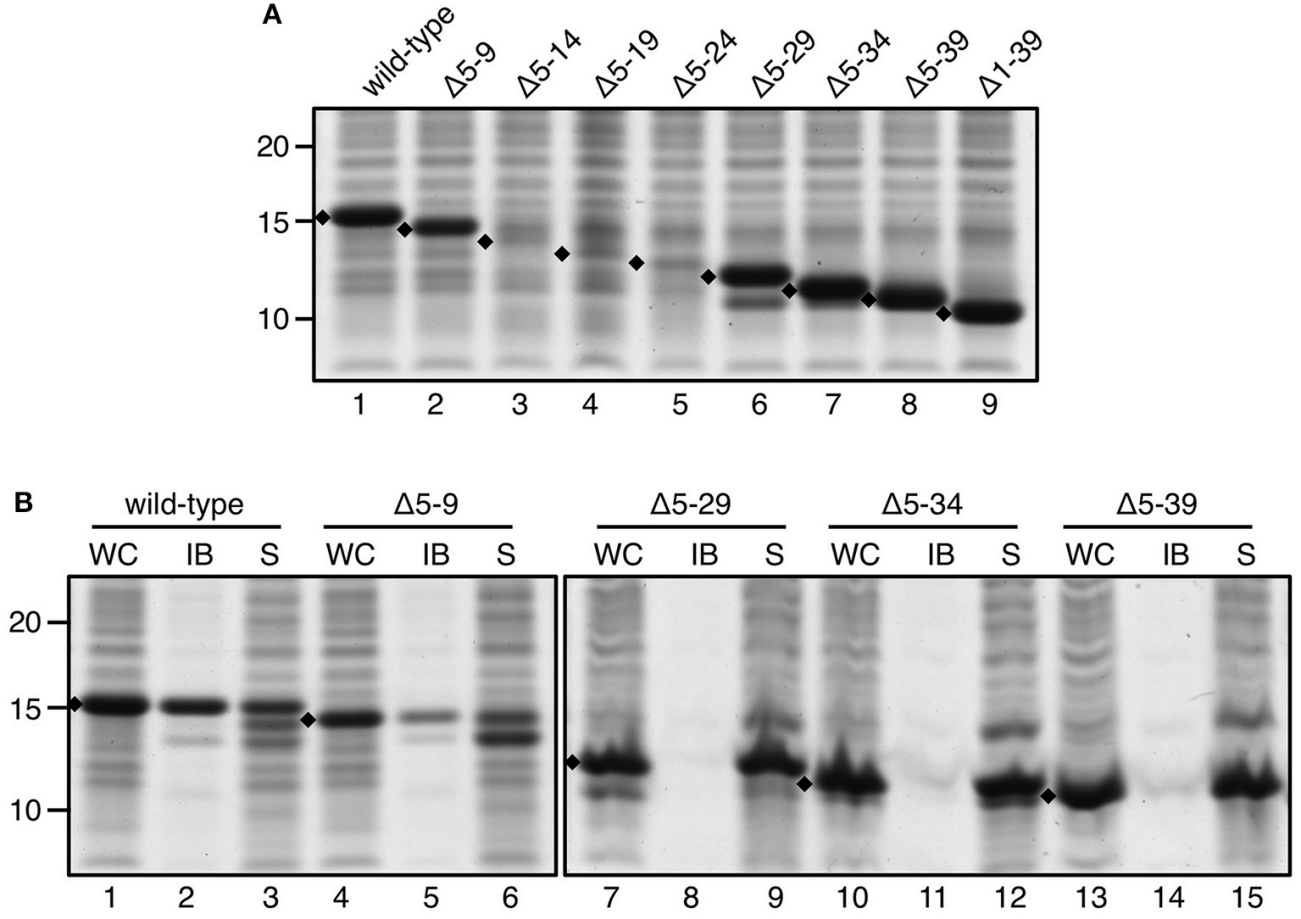
IB formation upon incremental deletion from the N-terminus of ssTorA. **(A)** SDS-PAGE/Coomassie staining analysis on cells overexpressing TrxA fused to wild-type ssTorA or an ssTorA variant carrying any of the indicated amino acid deletions. Expression of TrxA not fused to any ssTorA moiety is labeled Δ1–39. **(B)** IB-sedimentation assay as described in the legend to Figure 1 on cells expressing a selection of the ssTorA/TrxA fusions from *A*. Bands representing signal sequence/TrxA fusions of interest are indicated (♦). Molecular mass (kDa) markers are indicated at the left side of the panels.

To further narrow down the regions important for IB formation, we followed a strategy in which incremental parts of ssTorA in the ssTorA/TrxA construct were deleted, starting from the N- and C-terminus, respectively. The set of N-terminal deletion mutants started at position 5 to keep the NNN motif at position 2–4 intact for optimal expression. The number of deleted residues increased by 5 for each consecutive mutant (Supplementary Table 1). Remarkably, whereas fusions carrying deletions up to residue 9 or extending beyond residue 29 were efficiently expressed (Figure 5A, lanes 1–2, 6–9), versions with deletions Δ5–14, Δ5–19, and Δ5–24 were hardly detectable in whole cell lysates by SDS-PAGE (Figure 5A, lanes 3–5). To investigate whether the poor expression was due to problems at the mRNA level, we performed quantitative PCR using *trxA*-targeting primers to determine the levels of mRNA encoding the non-detectable ssTorA(Δ5–19)/TrxA vs. the strongly expressed ssTorA/TrxA and ssTorA(Δ5–34)/TrxA (Supplementary Figure 1). A considerable reduction in encoding mRNA was observed specifically for ssTorA(Δ5–19)/TrxA, which might suggest that deletions Δ5–14, Δ5–19 and Δ5–24 introduce mRNA instability, explaining their poor expression. The highly expressed constructs were analyzed for IB formation as before. In line with the above observation that the N-terminal part of ssTorA is dispensable for the production of IBs (see Figure 5A), insoluble expression was observed for ssTorA(Δ5–9)/TrxA (Figure 5B, lane 5). In contrast, fusions carrying deletions Δ5–29, Δ5–34 or Δ5–39 were soluble (Figure 5B, lanes 9,12,15). It follows that a region critical for the induction of IBs must be localized downstream of residue 9 and upstream of residue 29 of ssTorA.

**Figure 6:**
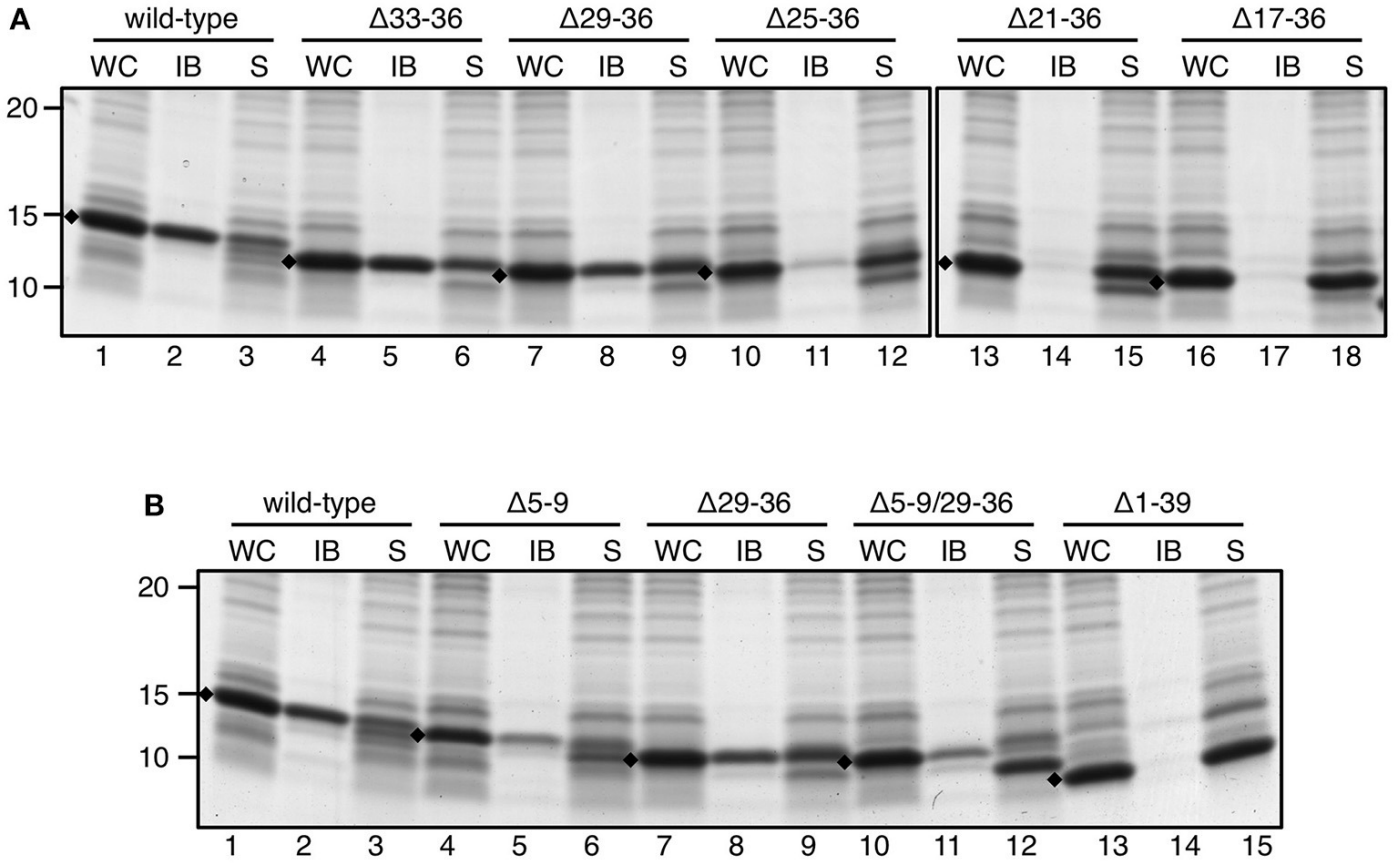
IB formation upon C-terminal and combined N/C-terminal deletions. **(A,B)** IB-sedimentation assay as described in the legend to Figure 1 on cells expressing TrxA fused to wild-type ssTorA or an ssTorA variant carrying any of the indicated amino acid deletions. Expression of TrxA not fused to any ssTorA moiety is labeled Δ1–39. Bands representing signal sequence/TrxA fusions of interest are indicated (♦). Molecular mass (kDa) markers are indicated at the left side of the panels.

A similar deletion strategy was applied on ssTorA but now starting from the C-terminus. Leaving the C-terminal ASA motif intact, we removed residues 36 to 17 by deleting three or four residues per consecutive mutant (Supplementary Table 1; Figure 6A). Efficient expression was now found for all ssTorA/TrxA mutants allowing analysis of IB formation. Deletions up to proline 29 of ssTorA (Δ29–36) did not interfere with IB formation (Figure 6A, lane 8) whereas derivatives with larger deletions were completely soluble (Figure 6A, lanes 11, 14, 17). Next, the largest N- and C-terminal deletions that still formed IBs were combined in one ssTorA/TrxA construct (Supplementary Table 1) and tested (Figure 6B). Indeed, ssTorA(Δ5–9/Δ29–36) still induced IB formation to a similar extent as ssTorA(Δ5–9) (Figure 6B, c.f. lane 11 and 5) but less efficiently than the intact ssTorA sequence (Figure 6B, lane 2). This suggests that residues 5–9 are not required for IB formation *per se*, but do enhance aggregation. Together, the data limit the region of ssTorA required for IB formation roughly to residues 10–28.

### Features of ssTorA Involved in IB Formation

Given the importance of residues 10–28 that roughly correspond to the hydrophobic H-domain of ssTorA (AA 14–32) (Cristobal et al., 1999), a potential scenario for the initiation of IB formation would involve hydrophobic interactions between neighboring ssTorA sequences or between ssTorA and fused cargo proteins. However, the hydrophobicity of ssTorA is relatively modest and similar or lower than that of other Tat signal sequences that did not form IBs (Tullman-Ercek et al., 2007) (see Figure 2). Alternatively, the tendency of signal sequence H-domains to attain an α-helical conformation was considered. Indeed, substitution of Ala16 in the core of ssTorA by a proline (A16P) prevented insoluble expression of ssTorA/TrxA (Supplementary Figure 3B, c.f. lanes 2 and 5), suggesting that α-helix formation is a prerequisite for IB formation. Interestingly, helical wheel representation of the ssTorA revealed a concentration of hydrophobic residues at one side of the helix (F7-F14-L18-L21) (Supplementary Figure 3A, *boxed*). Non-conservative substitution of either one (F14 > S14) or two (F7 > A7 + F14 > S14) of these residues reduced the amount of ssTorA/TrxA recovered in the insoluble cell fraction (Supplementary Figure 3C, lanes 5 and 8), suggesting the importance of this feature in IB formation. Possibly, the imposed clustering of hydrophobic residues nucleates the assembly of fusion proteins into IBs.

### Screening for Residues Critical for IB Formation

To identify residues important for IB induction in a more unbiased way, we developed a library screen with randomly mutagenized ssTorA (Supplementary Figure 4). Chloramphenicol acetyl transferase (CAT) was used as a fusion partner for the ssTorA library reasoning that ssTorA/CAT can only confer resistance toward the antibiotic chloramphenicol when expressed in soluble form (Maxwell et al., 1999). Hence, only cells with mutant ssTorA that has lost its capacity to deposit CAT in inclusion bodies are expected to grow on chloramphenicol containing plates.

First, we confirmed that CAT is expressed in IBs when fused to wild-type ssTorA (Supplementary Figure 5, lane 6), whereas the majority of fusion protein remains soluble when a non-functional variant [ssTorA(N′)] is used (Supplementary Figure 5, lane 2). Next, we determined the conditions that allow on-plate discrimination between cells expressing the soluble and insoluble CAT fusions. To ensure optimal protein expression and IB formation cells were first grown and induced in liquid culture before being spread on LB-plates containing chloramphenicol for overnight selection of resistant cells (Supplementary Figure 4). When testing cells expressing either insoluble ssTorA/CAT or soluble ssTorA(N′)/CAT on plates without the antibiotic, growth was observed in both cases (Supplementary Figure 5B). In contrast, growth on LB-agar supplemented with 30 μg/ml chloramphenicol yielded colonies only for the cells expressing the soluble ssTorA(N′)/CAT fusion (Supplementary Figure 5B), providing proof of concept for the screening assay.

**Figure 7:**
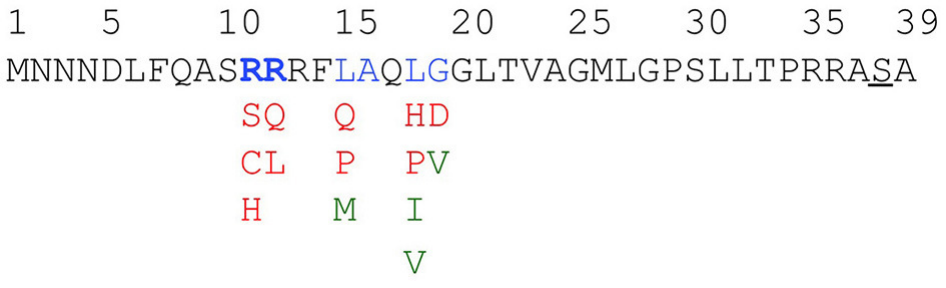
Favored and non-favored substitutions for interference with IB tag functionality. Amino acids in the ssTorA tag predicted to be important for IB formation—as based on data displayed in Supplementary Figure 6—are colored blue. For each of these amino acids, substituting residues potentially interfering with ssTorA tag functionality—as based on data displayed in Supplementary Figure 7—are colored red. Substituting residues predicted to keep tag functionality unaffected are displayed in green.

Next, we fused a library of randomly mutagenized ssTorA fragments to CAT and used the on-plate chloramphenicol survival assay to select for cells that express soluble ssTorA/CAT mutants. Next-generation sequencing was used to define the mutations in individual ssTorA encoding DNA fragments (Supplementary Figure 4). In our analysis we focused on a 138 bp sequence including 21 upstream bases that contain the ribosome binding site. Interestingly, mutation hotspots were identified in DNA obtained from chloramphenicol resistant cells for the R11, R12, L15, L18, and G19 codons (Supplementary Figure 6). In all these cases the mutations were in the second nucleotide of the codons, consequently resulting in amino acid substitutions. Importantly, the detected bias against mutagenesis in the Shine-Dalgarno sequence, which is needed for efficient expression of ssTorA/CAT, is consistent with the validity of the screening procedure. Taken together, we identified R11, R12, L15, L18, and G19 of ssTorA as potentially critical residues for IB formation (Figure 7).

A bias for specific changes in the mutational hotspots suggested amino acid substitutions potentially responsible for enhanced ssTorA/CAT solubility (Supplementary Figure 7). To further validate the screening procedure and analyze the importance of R11, R12, L15, L18, and G19 these residues were individually replaced with the selected residues (Figure 7). Using the same conditions as above, all mutants indeed induced a chloramphenicol-resistant phenotype in *E. coli* on plate (Supplementary Figure 8), implying expression of ssTorA/CAT in a (partially) soluble fashion.

**Figure 8:**
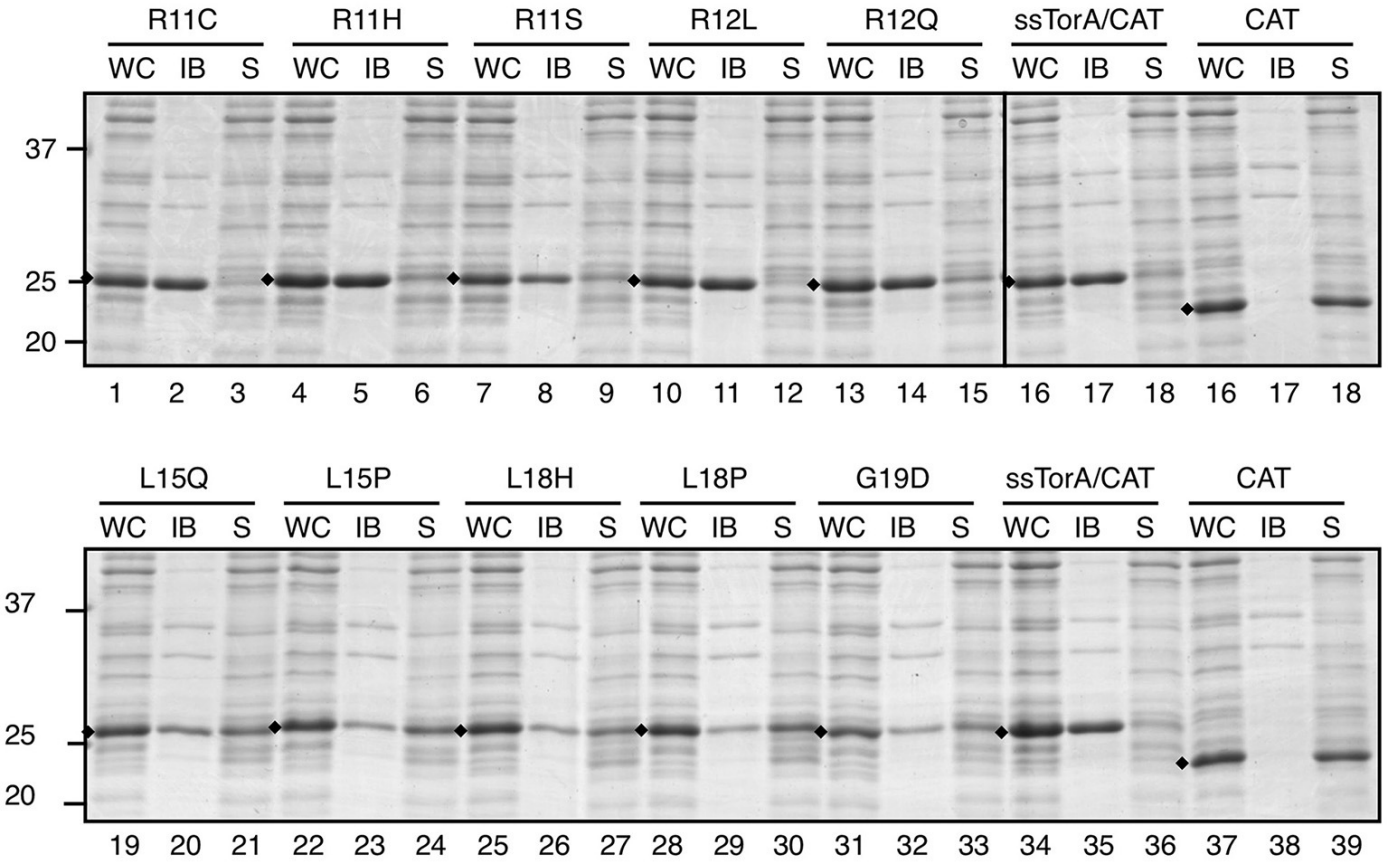
Influence of potential solubilizing substitutions on IB formation of ssTorA/CAT. IB-sedimentation assay as described in the legend to Figure 1 on cells expressing ssTorA/CAT derivatives carrying the indicated single amino acid substitutions in the ssTorA moiety. ssTorA/CAT carrying wild-type ssTorA and CAT lacking ssTorA (*CAT*) were analyzed in parallel as controls. Bands representing fusion proteins of interest are indicated (♦). Molecular mass (kDa) markers are indicated at the left side of the panels.

Next, we analyzed the solubility of the mutant fusion proteins using the sedimentation assay (Figure 8). Impaired IB formation compared to non-mutated ssTorA/CAT (Figure 8, lanes 19–33) could be confirmed for L15, L18, and G19 mutants, which were recovered largely in the soluble fraction (Figure 8, lanes 19–33). In contrast, changes at R11 and R12 did not clearly affect IB formation despite the use of rather non-conserved substitutions (Figure 8, c.f. lanes 1–15 and 16–18). As a control, we also substituted L15, L18, and G19 with residues that were expected not to interfere with the functionality of the tag based on the Next-generation sequencing analysis (Figure 7; Supplementary Figure 7). Indeed, the resulting ssTorA/CAT fusions (M15, Q17, I18, V18, and G19) accumulated almost exclusively in IBs (Supplementary Figure 9A). Together, these data suggest that our method effectively discriminates between solubilizing and non-solubilizing mutations in ssTorA/CAT.

To exclude that our findings were specific for fusions to CAT, the same substitutions were tested in the context of a second cargo protein, TrxA, with very similar results (Supplementary Figures 9B, 10). Whereas mutation of positions R11 and R12 did not affect IB formation (Supplementary Figure 10, c.f. lanes 1–15 and 16–18), substitutions at positions 15, 18, or 19 resulted in elevated levels of soluble ssTorA/TrxA (Supplementary Figure 10, c.f. lanes 19–33 and 37–39) except for M15, Q17, I18, V18, and G19 (Supplementary Figure 9B). In conclusion, our combined mutagenesis and screening approach identified five potentially important residues for ssTorA-mediated IB formation. Three of these (L15, L17, and G19) were confirmed to be critical using IB sedimentation analysis.

### Tandem-Fused Truncated ssTorA Enhance IB Formation

**Figure 9:**
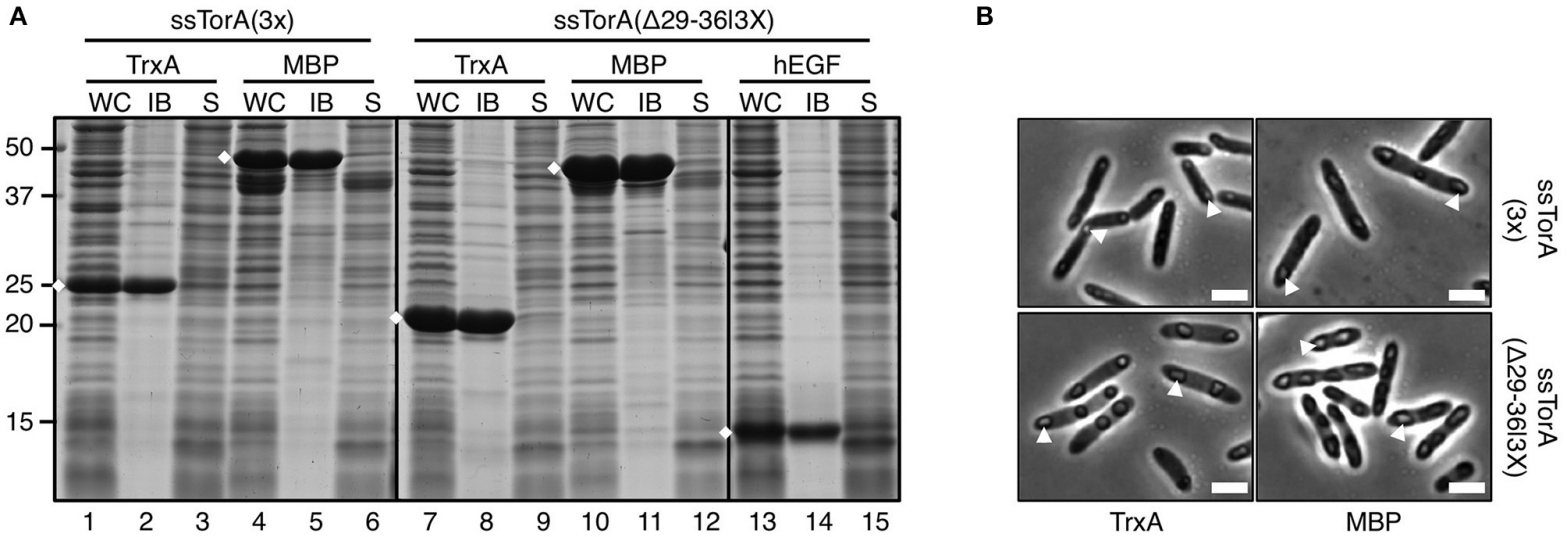
IB formation upon fusion to tandem-fused truncated ssTorA. **(A)** IB-sedimentation assay as described in the legend to Figure 1 on cells expressing TrxA, MBP, or hEGF fused to either ssTorA(3X) or ssTorA (Δ29–36|3X) as indicated. Bands representing the fusions of interest are indicated (♦). **(B)** Phase-contrast microscopy on a selection of cells from *A* as indicated. Examples of IBs are indicated with white arrowheads. Scale bar 2 μm.

Previously, we have shown that suboptimal deposition of cargo proteins in IBs could be enhanced by fusing three ssTorA in tandem [ssTorA(3X)] (Jong et al., 2017). Since for biotechnological applications a short IB tag is preferred, a triple tag of reduced length was made by linking three ssTorA sequences lacking residues 29–36, which appeared dispensable for IB formation (see under Regions Critical for IB Formation) (Supplementary Table 1). To test ssTorA(Δ29/36|3x) it was not only fused to TrxA but also to another highly soluble *E. coli* protein, MBP, and to a small recombinant protein of human origin, hEGF. All fusion proteins were almost exclusively detected in the insoluble pellet upon sedimentation of IBs (Figure 9A, lanes 8, 11, 14), demonstrating that ssTorA(Δ29/36|3x) indeed functions as an IB formation tag with a higher efficiency than single ssTorA(Δ29/36). Surprisingly, when compared side-by-side to TrxA and MBP carrying full-length ssTorA(3X), fusions to the new truncated tag were expressed at a higher level (Figure 9A, c.f. lanes 7 and 1; c.f. lanes 10 and 4) and yielded higher amounts of fusion protein in IBs (Figure 9A, c.f. lanes 8 and 2; c.f. lanes 11 and 5). Consistently, analysis of cells by phase contrast microscopy showed more pronounced IB structures for the fusions carrying the truncated triple tag (Figure 9B). To exclude that the altered (*E. coli* optimized) codon usage of the synthetic ssTorA(Δ29/36|3x) coding sequence is responsible for the effect, ssTorA(Δ29/36|3x; NO) with a codon usage identical to that of the full length ssTorA(3X) (Supplementary Figure 2A) was shown to induce similar elevated expression and IB yields (Supplementary Figure 2B). In conclusion, the shortened triple fusion tag has improved IB-formation properties compared to the original ssTorA(3X) sequence.

## Discussion

Previously, we have shown that fusion of heterologous protein to the signal sequence of TorA did not promote targeting but rather deposition of the fusion proteins in insoluble aggregates (Jong et al., 2017). In fact, ssTorA proved to be useful as a small tag for robust inclusion body formation of even highly soluble proteins. In this study we characterized the requirements for inclusion body induction leading to a shortened and improved tag.

As ssTorA is a Tat signal sequence we considered the possibility that targeting of the fusion protein to the Tat translocon combined with its translocation incompetence might increase the local concentration at the membrane and nucleate aggregation. However, neither the invariant twin arginine (RR) motif for Tat signal sequences nor the presence of a functional Tat translocation machinery in the bacterial inner membrane appeared critical for IB formation (see Figure 1). Additionally, eleven other Tat signal sequences of varying architecture did not induce aggregation, except for ssCueO that displays relatively low similarity to ssTorA. IB formation appeared also unrelated to the existence of a cognate cytosolic chaperone since ssDmsA, which also binds a chaperone and is very similar to ssTorA in function and length (Shanmugham et al., 2012), does not produce aggregates of fused cargo protein (see Figure 2). This all suggests that the IB formation properties are contained in unique sequence characteristics of ssTorA rather than being related to its physiological role.

Using a systematic deletion approach (Figures 4, 6) we could limit the region of ssTorA predominantly responsible for IB formation to residues 10–28. Yet, the presence of residues 2–9 appeared to enhance aggregation efficiency probably by facilitating optimal translation initiation as a consequence of high A/T codon usage (Qing et al., 2003). Based on the deletion analysis we designed a truncated ssTorA triple fusion tag lacking residues 29–36 [ssTorA(Δ29/36|3x)]. The truncated triple tag proved to be superior to the original triple tag comprising three full-length copies of ssTorA (Jong et al., 2017) with respect to fusion protein yield and IB formation (see Figure 9). Interestingly, the deletion removes two prolines and two arginines per ssTorA copy, residues that have been reported to promote stalling of nascent polypeptide chains in the ribosomal exit tunnel (Elgamal et al., 2014; Woolstenhulme et al., 2015).

Unbiased genetic screening of a library of randomly mutagenized ssTorA sequences for reduced aggregation properties using a assay allowed us to pinpoint residues that are critical for induction of IB formation. Importantly, the location of these residues was in agreement with the essential region for IB formation as identified by deletion mutagenesis (see Figure 6). Moreover, three of the five residues were also demonstrated to be critical for IB formation upon growth in culture (see Figure 8). This apparent discrepancy between the sedimentation procedure and the on-plate chloramphenicol-survival assay may be due to faster cell growth in the former, slightly favoring IB formation of ssTorA fusions with close-to-threshold aggregation properties. Although the focus was on our IB-formation tag, the presented screening methodology may be more generally applicable for studying protein aggregation in microbial model systems.

Prevailing mechanisms for the initiation of aggregation involve the exposition of hydrophobic stretches that interact with neighboring protein sequences and/or more specific stacking of β-strands that nucleates the formation of amyloid-like cross β-structures (Fink, 1998; Garcia-Fruitos et al., 2011; Bednarska et al., 2013). However, ssTorA neither possesses strong hydrophobicity (Tullman-Ercek et al., 2007) nor has predicted propensity to form β-aggregates (Fernandez-Escamilla et al., 2004) (data not shown). Formation of an amyloid structure by a peptide that maintained its α-helical conformation was recently presented (Tayeb-Fligelman et al., 2017), but it is hard to envision such a model for ssTorA when fused to cargos of substantial size. We did, however, identify a hydrophobic patch that may form when the ssTorA attains an α-helical conformation. Introduction of a proline indeed impaired IB formation (see Supplementary Figure 3) and disruptive proline substitutions (L15P and L18P) were also selected in our unbiased screening assay, suggesting the importance of helix formation (see Figures 7, 8). Intermolecular interactions between the hydrophobic patches on respective ssTorAs are expected to be insufficient to nucleate IB formation as only two molecules would be able to associate in this fashion. On the other hand, this could be sufficient to hamper folding of the juxtaposed cargo proteins, leading to exposure of aggregation prone sequences. Consistent with such an indirect mechanism, the efficiency of IB formation varies depending on the nature of the fused cargo protein (e.g., see Supplementary Figure 9).

Co-overexpression of ssTorA fusions with its cognate cytosolic chaperone TorD prevented IB-formation suggesting that TorD covers a critical aggregation feature (see Figure 3). Two-hybrid analysis indeed localized the signal sequence-binding site of a TorD family chaperone to the Tat-consensus sequence (see Figure 1A) and several residues downstream (Coulthurst et al., 2012), which is located in the region identified in this study as being critical for IB formation. Possibly, TorD has a similar role under physiological conditions, preventing aggregation of TMAO-reductase in the bacterial cytoplasm prior to translocation across the cell envelope (Hatzixanthis et al., 2005). Similarly, overexpression of DnaK was found to impair sedimentation of fusion proteins (see Figure 3) and a predicted binding site in ssTorA (residues 12–18) (Van Durme et al., 2009) appears to be included in the IB-formation motif. Conceivably, the motif must be exposed to trigger IB formation. Alternatively, considering the generic role of DnaK in protein folding (Kim et al., 2013), strong overexpression of ssTorA may sequester DnaK from the cytoplasm inducing aggregation of fused cargo proteins.

In conclusion, the data provided detailed insight into regions and residues of ssTorA that are critical for its functioning as an IB formation tag. Furthermore, this study yielded an improved truncated version of the ssTorA tag that may be exploited for insoluble protein production in biotechnological and biomedical applications.

## Data Availability Statement

The datasets generated for this study can be found in the personal repositories of the authors and are available to any qualified researcher upon request, subject to the signing of an MTA.

## Author Contributions

WJ and JL made major contributions to the conception and design of the study and writing of the manuscript. WJ, CH-J, WD, DV, J-WG, AA, WB, and JL contributed to the acquisition, analysis, or interpretation of the data. DV, AA, J-WG, and WB critically read the manuscript.

### Conflict of Interest

WJ, J-WG, and JL are involved in Abera Bioscience AB that aims to exploit the ssTorA inclusion body formation tag for biotechnology purposes. DV is employed at Xbrane Biopharma AB, a forerunner of Abera Bioscience AB. The remaining authors declare that the research was conducted in the absence of any commercial or financial relationships that could be construed as a potential conflict of interest.
