# Supplementary material for: Mutagenesis-Based Characterization and Improvement of a Novel Inclusion Body Tag
Source: Front Bioeng Biotechnol. 2020 Jan 10;7:442. doi: 10.3389/fbioe.2019.00442 (PMC6965018; doi:10.3389/fbioe.2019.00442)
Supplement: Supplementary file 1 [file Data_Sheet_1.PDF]

Supplementary Materials and Methods

Plasmid construction

Unless stated otherwise, proteins were expressed under control of a *tetA* promoter from vector pASK-IBA3 (IBA GmbH). Plasmids pIBA-TrxA, pIBA-ssTorA/TrxA, pIBA-ssTorA(3x)/TrxA and pIBA-ssTorA(3x)/MBP have been described previously (Jong et al. 2017). Plasmids for the overexpression of chloramphenicol acetyl transferase (CAT) (fusions) were made by PCR. To amplify the CAT-encoding region, pEH3-Hbp (Jong et al. 2017) was used as a template. To construct pIBA-CAT, the primers used were *Xba*I-RBS-CAT fw and *Hin*dIII-CAT rv. The resulting fragment was cloned into pIBA-TrxA using the *Xba*I/*Hin*dIII restriction sites, yielding pIBA-CAT. To construct pIBA-ssTorA/CAT, the primers used were *Nhe*I-CAT fw and *Hin*dIII-CAT rv. The PCR product was cloned into the *Nhe*I/*Hin*dIII sites of pIBA-ssTorA/TrxA, yielding pIBA-ssTorA/CAT.

pIBA-based constructs encoding fusions between TrxA and the Tat-signal sequences ssAmiC, ssCueO, ssDmsA, ssFdnG, ssHyaA, ssHybO, ssNapA, ssNapG, ssWcaM, ssybaK, and ssYagT were generated by PCR. *E. coli* MG1655 genomic DNA was used as a template in combination with a specific fw and rv primer set for each individual signal sequence/TrxA construct (see Table S3). The respective PCR fragments were ligated into the *Xba*I/*Nhe*I sites of pIBA-ssTorA/TrxA, replacing the ssTorA-encoding sequence by those of the alternative Tat-signal sequences.

pIBA-based constructs encoding fusions carrying single amino acid substitutions in ssTorA were created by overlap-extension PCR using the mutagenesis primers listed in Table S3 and flanking primers IBA_*Xba*I_ssTorA fw and ASAA_ssTorA rv. The resulting PCR fragments were ligated into the *Xba*I/*Nhe*I sites of pIBA-ssTorA/TrxA and pIBA-ssTorA/CAT to replace the wild-type ssTorA-encoding sequence by the mutant DNA. Plasmid pIBA-ssTorA(RR/AA) was created in similar fashion using the mutagenesis primers ssTorA(RR/AA) fw and ssTorA(RR/AA) rv. Moreover, pIBA-ssTorA(F7A-F14S)/TrxA was created by using mutagenesis primers ssTorA(F7A-F14S) fw and ssTorA(F7A-F14S) rv

To create substitution mutant pIBA-ssTorA(NNN/KTK)/TrxA a PCR was carried out using pIBA-ssTorA/TrxA as a template and the primers ssTorA(NNN/KTK) fw and ASAA_ssTorA rv. The resulting fragment was cloned into the *Xba*I/*Nhe*I sites of pIBA-ssTorA/TrxA to obtain the final plasmid. Similarly, pIBA-ssDmsA(KTK/NNN)/TrxA was created using pIBA-ssDmsA/TrxA as the template and the primers ssDmsA(KTK/NNN) fw and ssDmsA rv. The PCR fragment was ligated into the *Xba*I/*Nhe*I sites of pIBA-ssDmsA/TrxA to obtain pIBA-ssDmsA(KTK/NNN)/TrxA. To create pIBA-ssTorA(C')/TrxA encoding an N-terminally truncated version of ssTorA, PCR was performed to amplify *sstorA* using truncating primer ssTorA(C') fw in combination with ASAA_ssTorA rv. The resulting fragment was cloned into the *Xba*I/*Nhe*I sites of pIBA-ssTorA/TrxA to yield the truncated variant. Using the same strategy pIBA(C'+NNN)/TrxA was made using truncating primer ssTorA(C'+NNN) fw. Again, similar methodology was used for creating truncated mutants of pIBA-ssTorA/TrxA carrying deletions Δ5-9, Δ5-14, Δ5-19, Δ5-24, Δ5-29, Δ5-34 and Δ5-39 utilizing the corresponding fw mutagenesis primers listed in Table S3. Plasmid pIBA-ssTorA(N')/TrxA, encoding a C-terminally truncated version of ssTorA, was made by amplification of *sstorA* using primer IBA_*Xba*I_TorA fw and truncating primer ssTorA(N') rv. The resulting fragment was cloned into the *Xba*I/*Nhe*I sites of pIBA-ssTorA/TrxA to yield the truncated mutant

plasmid. The same fragment was ligated into the *Xba*I/*Nhe*I sites of pIBA-ssTorA/CAT to yield ssTorA(N')/CAT. Using a similar truncation approach mutants of pIBA-ssTorA/TrxA carrying deletions Δ17-36, Δ21-36, Δ25-36, Δ29-36 and Δ33-36 were created by using the corresponding rv mutagenesis primers listed in Table S3. To construct a pIBA-ssTorA/TrxA variant carrying the deletions Δ5-9 and Δ29-36 combined, PCR was performed using pIBA-ssTorA/TrxA as the template and the primers ssTorA(Δ5-9) fw and ssTorA(Δ29-36) rv. The resulting fragment was cloned into pIBA-ssTorA/TrxA using the *Xba*I/*Nhe*I restriction sites, yielding pIBA-ssTorA(Δ5-9/Δ29-36)/TrxA.

To create pIBA-ssTorA(Δ29-36|3x)/TrxA carrying an *E. coli* codon-optimized version of the ssTorA(Δ29-36|3x) coding sequence (see Fig. S2A) a synthetic DNA fragment was ordered (IDT; Coralville, Iowa, US) encoding ssTorA(Δ29-36|3x) flanked by the upstream TAACGAGGCAAAAA sequence and *Xba*I/*Nhe*I sites as present in pIBA-ssTorA/TrxA. The fragment was inserted into the *Xba*I/*Nhe*I sites of pIBA-ssTorA/TrxA using In-Fusion cloning (Takara Bio Inc.), yielding pIBA-ssTorA(Δ29-36|3x)/TrxA. Using the same procedure, the fragment was inserted into pIBA-ssTorA/MBP and pIBA-ssTorA/hEGF (Jong et al. 2017) to create pIBA-ssTorA(Δ29-36|3x)/MBP and pIBA-ssTorA(Δ29-36|3x)/hEGF, respectively. A non-codon optimized version of pIBA-ssTorA(Δ29-36|3x)/TrxA was created by PCR as follows. First, a ssTorA(Δ29-36|3x)-encoding fragment with flanking *Spe*I/*Nhe*I sites was was amplified using pIBA-ssTorA(Δ29-36|3x)/TrxA as a template and the primers *Spe*I_ssTorA rep fw and *Nhe*I_ssTorA(Δ29-36) rv. The resulting fragment was ligated into the *Nhe*I site of pIBA-ssTorA(Δ29-36)/TrxA, yielding pIBA-ssTorA(Δ29-36|2x)/TrxA. Subsequently, the same PCR fragment was ligated into pIBA-ssTorA(Δ29-36|2x)/TrxA to yield pIBA-ssTorA(Δ29-36|3x;NO)/TrxA. This plasmid encodes a version of ssTorA(Δ29-36|3x) that carries an additional AS linker between the individual ssTorA(Δ29-36) segments compared to the codon optimized version described above (see Fig. S2A).

Plasmids pOFX-tac1 and pOFX-tac-DnaK/DnaJ1 have been described before (Castanie et al. 1997). To construct plasmid pOFX-tac-MBP, encoding MBP without its native signal sequence, a PCR was carried out using pIBA-MBP (Jong et al. 2017) as a template and the primers *Nde*I MBP fw and *Bam*HI rv. The resulting fragment was cloned into pOFX-tac-DnaK/DnaJ1 using the *Nde*I/*Bam*HI sites, yielding pOFX-tac-MBP. pLysTac-TorD is derived from plasmid pLysTac. This plasmid (gift Y.Bollen, Vrije Universiteit Amsterdam) is a derivative from pACT3 having the Shine-Dalgarno sequence, multiple cloning site and T7 terminator from pET3c inserted between the p15a origin and *lac* operator. The *Nde*I/*Bam*HI fragment of *E. coli* MG1655 TorD-encoding plasmid pTorD-17 (gift Y.Bollen, Vrije Universiteit Amsterdam) was subcloned into pLysTac to create pLysTac-TorD.

References

Castanie, M. P., H. Berges, J. Oreglia, M. F. Prere and O. Fayet (1997). "A set of pBR322-compatible plasmids allowing the testing of chaperone-assisted folding of proteins overexpressed in Escherichia coli." Anal Biochem **254**(1): 150-152.

Jong, W. S., D. Vikstrom, D. Houben, H. B. van den Berg van Saparoea, J. W. de Gier and J. Luirink (2017). "Application of an E. coli signal sequence as a versatile inclusion body tag." Microb Cell Fact **16**(1): 50.

Supplementary Table 1. Signal peptides investigated in this study

Name	Amino acid sequence
ssTorA (native)	MNNNDLFQASRRRFLAQLGGLTVAGMLGPSLLTPRRATA
ssTorA	MNNNDLFQASRRRFLAQLGGLTVAGMLGPSLLTPRRASA
ssTorA(NNN/KTK)	M**KTK**DLFQASRRRFLAQLGGLTVAGMLGPSLLTPRRASA
ssTorA(N′)	MNNNDLFQASRRR-----------------------ASA
ssTorA(C′)	M------------FLAQLGGLTVAGMLGPSLLTPRRASA
ssTorA(C′+NNN)	MNNN---------FLAQLGGLTVAGMLGPSLLTPRRASA
ssTorA(Δ5-9)	MNNN-----SRRRFLAQLGGLTVAGMLGPSLLTPRRASA
ssTorA(Δ5-14)	MNNN----------LAQLGGLTVAGMLGPSLLTPRRASA
ssTorA(Δ5-19)	MNNN---------------GLTVAGMLGPSLLTPRRASA
ssTorA(Δ5-24)	MNNN--------------------GMLGPSLLTPRRASA
ssTorA(Δ5-29)	MNNN-------------------------SLLTPRRASA
ssTorA(Δ5-34)	MNNN------------------------------RRASA
ssTorA(Δ5-39)	MNNN-----------------------------------
ssTorA(Δ33-36)	MNNNDLFQASRRRFLAQLGGLTVAGMLGPSLL----ASA
ssTorA(Δ29-36)	MNNNDLFQASRRRFLAQLGGLTVAGMLG--------ASA
ssTorA(Δ25-36)	MNNNDLFQASRRRFLAQLGGLTVA------------ASA
ssTorA(Δ21-36)	MNNNDLFQASRRRFLAQLGG----------------ASA
ssTorA(Δ17-36)	MNNNDLFQASRRRFLA--------------------ASA
ssTorA(Δ5-9/Δ29-36)	MNNN-----SRRRFLAQLGGLTVAGMLG--------ASA
ssTorA(RR/AA)	MNNNDLFQAS**AA**RFLAQLGGLTVAGMLGPSLLTPRRASA
ssTorA(A16P)	MNNNDLFQASRRRFL**P**QLGGLTVAGMLGPSLLTPRRASA
ssTorA(F14S)	MNNNDLFQASRRR**S**LAQLGGLTVAGMLGPSLLTPRRASA
ssTorA(F7A+F14S)	MNNNDL**A**QASRRR**S**LAQLGGLTVAGMLGPSLLTPRRASA
ssTorA(R11C)	MNNNDLFQAS**C**RRFLAQLGGLTVAGMLGPSLLTPRRASA
ssTorA(R11H)	MNNNDLFQAS**H**RRFLAQLGGLTVAGMLGPSLLTPRRASA
ssTorA(R11S)	MNNNDLFQAS**S**RRFLAQLGGLTVAGMLGPSLLTPRRASA
ssTorA(R12L)	MNNNDLFQASR**L**RFLAQLGGLTVAGMLGPSLLTPRRASA
ssTorA(R12Q)	MNNNDLFQASR**Q**RFLAQLGGLTVAGMLGPSLLTPRRASA
ssTorA(L15Q)	MNNNDLFQASRRRF**Q**AQLGGLTVAGMLGPSLLTPRRASA
ssTorA(L15P)	MNNNDLFQASRRRF**P**AQLGGLTVAGMLGPSLLTPRRASA
ssTorA(L18H)	MNNNDLFQASRRRFLAQ**H**GGLTVAGMLGPSLLTPRRASA
ssTorA(L18P)	MNNNDLFQASRRRFLAQ**P**GGLTVAGMLGPSLLTPRRASA
ssTorA(G19D)	MNNNDLFQASRRRFLAQL**D**GLTVAGMLGPSLLTPRRASA
ssTorA(L15M)	MNNNDLFQASRRRF**M**AQLGGLTVAGMLGPSLLTPRRASA
ssTorA(Q17E)	MNNNDLFQASRRRFLA**E**LGGLTVAGMLGPSLLTPRRASA
ssTorA(L18I)	MNNNDLFQASRRRFLAQ**I**GGLTVAGMLGPSLLTPRRASA
ssTorA(L18V)	MNNNDLFQASRRRFLAQ**V**GGLTVAGMLGPSLLTPRRASA
ssTorA(G19V)	MNNNDLFQASRRRFLAQL**V**GLTVAGMLGPSLLTPRRASA
ssTorA(3x)	MNNNDLFQASRRRFLAQLGGLTVAGMLGPSLLTPRRASMNNNDLFQASRRRFLAQLGGLTVAGMLGPSLLTPRRASMNNNDLFQASRRRFLAQLGGLTVAGMLGPSLLTPRRASA
ssTorA(Δ29-36|3x)	MNNNDLFQASRRRFLAQLGGLTVAGMLG----------MNNNDLFQASRRRFLAQLGGLTVAGMLG----------MNNNDLFQASRRRFLAQLGGLTVAGMLG--------ASA
ssTorA	MNNNDLFQASRRRFLAQLGGLTVAGMLGPSLLTPRRASA
ssAmiC	MSGSNTAISRRRLLQGAGAMWLLSVSQVSASA
ssCueO	MQRRDFLKYSVALGVASALPLWSRAASA
ssFdnG	MDVSRRQFFKICAGGMAGTTVAALGFAPKQASA
ssHyaA	MNNEETFYQAMRRQGVTRRSFLKYCSLAATSLGLGAGMAPKIASA
ssHybO	MTGDNTLIHSHGINRRDFMKLCAALAATMGLSSKASA
ssNapA	MKLSRRSFMKANAVAAAAAAAGLSVPGVASA
ssNapG	MSRSAKPQNGRRRFLRDVVRTAGGLAAVGVALGLQQQTASA
ssWcaM	MPFKKLSRRTFLTASSALAFLHTPFASA
ssYcbK	MDKFDANRRKLLALGGVALGAAILPTPASA
ssYagT	MSNQGEYPEDNRVGKHEPHDLSLTRRDLIKVSAATAATAVVYPHSTASA
ssDmsA	MKTKIPDAVLAAEVSRRGLVKTTAIGGLAMASSALTLPFSRIASA
ssDmsA(KTK/NNN)	M**NNN**IPDAVLAAEVSRRGLVKTTAIGGLAMASSALTLPFSRIASA

Supplementary Table 2. Plasmids used in this study

Plasmid name	Description	Reference
pIBA-TrxA	Expression vector pASK-IBA3; *tetA* promoter + *trxA*	(Jong et al. 2017)
pIBA-ssTorA/TrxA	pIBA + *sstorA/trxA*	(Jong et al. 2017)
pIBA-ssAmiC/TrxA	pIBA + *ssamiC/trxA*	This work
pIBA-ssCueO/TrxA	pIBA + *sscueO/trxA*	This work
pIBA-ssDmsA/TrxA	pIBA + *ssdmsA/trxA*	This work
pIBA-ssFdnG/TrxA	pIBA + *ssfdnG/trxA*	This work
pIBA-ssHyaA/TrxA	pIBA + *sshyaA/trxA*	This work
pIBA-ssHybO/TrxA	pIBA + *sshybO/trxA*	This work
pIBA-ssNapA/TrxA	pIBA + *ssnapA/trxA*	This work
pIBA-ssNapG/TrxA	pIBA + *ssnapG/trxA*	This work
pIBA-ssWcaM/TrxA	pIBA + *sswcaM/trxA*	This work
pIBA-ssYbaK/TrxA	pIBA + *ssybaK/trxA*	This work
pIBA-ssYagT/TrxA	pIBA + *ssyagT/trxA*	This work
pIBA-ssTorA(NNN/KTK)/TrxA	pIBA-ssTorA/TrxA encoding NNN/KTK substitution	This work
pIBA-ssDmsA(KTK/NNN)/TrxA	pIBA-ssDmsA/TrxA encoding KTK/NNN substitution	This work
pIBA-ssTorA(N')/TrxA	pIBA-ssTorA/TrxA encoding N' truncation	This work
pIBA-ssTorA(C')/TrxA	pIBA-ssTorA/TrxA encoding C' truncation	This work
pIBA-ssTorA(C'+NNN)/TrxA	pIBA-ssTorA/TrxA encoding C' +NNN truncation	This work
pIBA-ssTorA(Δ5-9)/TrxA	pIBA-ssTorA/TrxA encoding Δ5-9 truncation	This work
pIBA-ssTorA(Δ5-14)/TrxA	pIBA-ssTorA/TrxA encoding Δ5-14 truncation	This work
pIBA-ssTorA(Δ5-19)/TrxA	pIBA-ssTorA/TrxA encoding Δ5-19 truncation	This work
pIBA-ssTorA(Δ5-24)/TrxA	pIBA-ssTorA/TrxA encoding Δ5-24 truncation	This work
pIBA-ssTorA(Δ5-29)/TrxA	pIBA-ssTorA/TrxA encoding Δ5-29 truncation	This work
pIBA-ssTorA(Δ5-34)/TrxA	pIBA-ssTorA/TrxA encoding Δ5-34 truncation	This work
pIBA-ssTorA(Δ5-39)/TrxA	pIBA-ssTorA/TrxA encoding Δ5-39 truncation	This work
pIBA-ssTorA(Δ33-36)/TrxA	pIBA-ssTorA/TrxA encoding Δ33-36 truncation	This work
pIBA-ssTorA(Δ29-36)/TrxA	pIBA-ssTorA/TrxA encoding Δ29-36 truncation	This work
pIBA-ssTorA(Δ25-36)/TrxA	pIBA-ssTorA/TrxA encoding Δ25-36 truncation	This work

Table continues on next page

Plasmid name	Description	Reference
pIBA-ssTorA(Δ21-36)/TrxA	IBA-ssTorA/TrxA encoding Δ21-36 truncation	This work
pIBA-ssTorA(Δ17-36)/TrxA	pIBA-ssTorA/TrxA encoding Δ17-36 truncation	This work
pIBA-ssTorA(Δ5-9/Δ29-36)/TrxA	pIBA-ssTorA/TrxA encoding Δ5-9/Δ29-36 truncation	This work
pIBA-ssTorA(3x)/TrxA	pIBA + *sstorA(3X)/trxA*	(Jong et al. 2017)
pIBA-ssTorA(Δ29-36|3x)/TrxA	pIBA-ssTorA(3X)/TrxA encoding Δ29-36 truncation	This work
pIBA-ssTorA(3x)/MBP	pIBA + *sstorA(3X)/malE*	This work
pIBA-ssTorA(Δ29-36|3x)/MBP	pIBA + *sstorA(3X)/malE* encoding Δ29-36 truncation	(Jong et al. 2017)
pIBA-ssTorA(Δ29-36|3x)/hEGF	pIBA + *sstorA(3X)/hegF* encoding Δ29-36 truncation	This work
pIBA-ssTorA(RR/AA)/TrxA	pIBA-ssTorA/TrxA encoding RR/AA substitution	This work
pIBA-ssTorA(A16P)/TrxA	pIBA-ssTorA/TrxA encoding A16P substitution	This work
pIBA-ssTorA(F14S)/TrxA	pIBA-ssTorA/TrxA encoding F14S substitution	This work
pIBA-ssTorA(F7A+F14S)/TrxA	pIBA-ssTorA/TrxA encoding F7A+F14S substitution	This work
pIBA-ssTorA(R11C)/TrxA	pIBA- ssTorA/TrxA encoding R11C substitution	This work
pIBA-ssTorA(R11H)/TrxA	pIBA-ssTorA/TrxA encoding R11H substitution	This work
pIBA-ssTorA(R11S)/TrxA	pIBA-ssTorA/TrxA encoding R11S substitution	This work
pIBA-ssTorA(R12L)/TrxA	pIBA-ssTorA/TrxA encoding R12L substitution	This work
pIBA-ssTorA(R12Q)/TrxA	pIBA-ssTorA/TrxA encoding R12Q substitution	This work
pIBA-ssTorA(L15Q)/TrxA	pIBA-ssTorA/TrxA encoding L15Q substitution	This work
pIBA-ssTorA(L15P)/TrxA	pIBA-ssTorA/TrxA encoding L15P substitution	This work
pIBA-ssTorA(L18H)/TrxA	pIBA-ssTorA/TrxA encoding L18H substitution	This work
pIBA-ssTorA(L18P)/TrxA	pIBA-ssTorA/TrxA encoding L18P substitution	This work
pIBA-ssTorA(G19D)/TrxA	pIBA-ssTorA/TrxA encoding G19D substitution	This work
pIBA-ssTorA(L15M)/TrxA	pIBA-ssTorA/TrxA encoding L15M substitution	This work
pIBA-ssTorA(Q17E)/TrxA	pIBA-ssTorA/TrxA encoding Q17E substitution	This work
pIBA-ssTorA(L18I)/TrxA	pIBA-ssTorA/TrxA encoding L18I substitution	This work
pIBA-ssTorA(L18V)/TrxA	pIBA-ssTorA/TrxA encoding L18V substitution	This work
pIBA-ssTorA(G19V)/TrxA	pIBA-ssTorA/TrxA encoding G19V substitution	This work
pIBA-CAT	pIBA + *cat*	This work
pIBA-ssTorA/CAT	pIBA + *sstorA/cat*	This work

Table continues on next page

Plasmid name	Description	Reference
pIBA-ssTorA(N')/CAT	pIBA-ssTorA/CAT encoding N' truncation	This work
pIBA-ssTorA(R11C)/CAT	pIBA-ssTorA/CAT encoding R11C substitution	This work
pIBA-ssTorA(R11H)/CAT	pIBA-ssTorA/CAT encoding R11H substitution	This work
pIBA-ssTorA(R11S)/CAT	pIBA-ssTorA/CAT encoding R11S substitution	This work
pIBA-ssTorA(R12L)/CAT	pIBA-ssTorA/CAT encoding R12L substitution	This work
pIBA-ssTorA(R12Q)/CAT	pIBA-ssTorA/CAT encoding R12Q substitution	This work
pIBA-ssTorA(L15Q)/CAT	pIBA-ssTorA/CAT encoding L15Q substitution	This work
pIBA-ssTorA(L15P)/CAT	pIBA-ssTorA/CAT encoding L15P substitution	This work
pIBA-ssTorA(L18H)/CAT	pIBA-ssTorA/CAT encoding L18H substitution	This work
pIBA-ssTorA(L18P)/CAT	pIBA-ssTorA/CAT encoding L18P substitution	This work
pIBA-ssTorA(G19D)/CAT	pIBA-ssTorA/CAT encoding G19D substitution	This work
pIBA-ssTorA(L15M)/CAT	pIBA-ssTorA/CAT encoding L15M substitution	This work
pIBA-ssTorA(Q17E)/CAT	pIBA-ssTorA/CAT encoding Q17E substitution	This work
pIBA-ssTorA(L18I)/CAT	pIBA-ssTorA/CAT encoding L18I substitution	This work
pIBA-ssTorA(L18V)/CAT	pIBA-ssTorA/CAT encoding L18V substitution	This work
pIBA-ssTorA(G19V)/CAT	pIBA-ssTorA/CAT encoding G19V substitution	This work
pOFX-tac1	Expression vector; *tac* promoter	(Castanie et al. 1997)
pOFX-tac-DnaK/DnaJ1	pOFX-tac + *dnaK/dnaJ*	(Castanie et al. 1997)
pOFX-tac-MBP	pOFX-tac + malE (encoding P0AEX9 AA 39-396)	This work
pLysTac-TorD	Expression vector; *tac* promoter + *torD*	This work

References

Castanie, M. P., H. Berges, J. Oreglia, M. F. Prere and O. Fayet (1997). "A set of pBR322-compatible plasmids allowing the testing of chaperone-assisted folding of proteins overexpressed in Escherichia coli." Anal Biochem **254**(1): 150-152.

Jong, W. S., D. Vikstrom, D. Houben, H. B. van den Berg van Saparoea, J. W. de Gier and J. Luirink (2017). "Application of an E. coli signal sequence as a versatile inclusion body tag." Microb Cell Fact **16**(1): 50.

Supplementary Table 3. Primers used in this study

Primer name	Primer sequence (5’ → 3’)
ssAmiC fw	cgtacgtctagataacgagggcaaaaaatgtcaggatccaacactgc
ssAmiC rv	gcatcggctagcactgacctgacttacgctca
ssCueO fw	cgtacgtctagataacgagggcaaaaaatgcaacgtcgtgatttcttaa
ssCueO rv	gcatcggctagctgcgcggctccacagcgg
ssDmsA fw	cgtacgtctagataacgagggcaaaaaatgaaaacgaaaatccctgatgcgg
ssDmsA rv	gcatcggctagcaatccgactaaaaggtaatgtta
ssFdnG fw	cgtacgtctagataacgagggcaaaaaatggacgtcagtcgcagac
ssFdnG rv	gcatcggctagcttgcttcggggcaaagcc
ssHyaA fw	cgtacgtctagataacgagggcaaaaaatgaataacgaggaaacattttac
ssHyaA rv	gcatcggctagcaatctttggtgccattcccg
ssHybO fw	cgtacgtctagataacgagggcaaaaaatgactggagataacaccctc
ssHybO rv	gcatcggctagctttgctacttaaccccatggtg
ssNapA fw	cgtacgtctagataacgagggcaaaaaatgaaactcagtcgtcgtagc
ssNapA rv	gcatcggctagcaacgcccggcacgctgag
ssNapG fw	cgtacgtctagataacgagggcaaaaaatgtcccggtcagcgaaacc
ssNapG rv	gcatcggctagcggtttgctgttgtaaccccag
ssWcaM fw	cgtacgtctagataacgagggcaaaaaatgccatttaaaaaactctccc
ssWcaM rv	gcatcggctagcgaaaggggtatggaggaagg
ssYcbK fw	cgtacgtctagataacgagggcaaaaaatggacaaattcgacgctaatc
ssYcbK rv	gcatcggctagcaggggtcggcaggatgg
ssYagT fw	cgtacgtctagataacgagggcaaaaaatgagcaaccaaggcgaatac
ssYagT rv	gcatcggctagccgtagaatgaggataaaccac
ssTorA(NNN/KTK) fw	cgtacgtctagataacgagggcaaaaaatgaaaacgaaagatctctttcaggcatcacg
IBA_XbaI_TorA fw	ctagtctagataacgagggcaaaaaatgaacaataacgatctctttc
ASAA_TorA rv	cgctgcgctagcacgtcgcggcgttaacaatg
ssDmsA(KTK/NNN) fw	cgtacgtctagataacgagggcaaaaaatgaacaataacatccctgatgcggtattggc
ssTorA(N’) rv	gcatcggctagcacgccgacgtgatgcctg
ssTorA(C’) fw	cgtacgtctagataacgagggcaaaaaaatgtttcttgcacaactcggcggcttaac
ssTorA(C’+NNN) fw	cgtacgtctagataacgagggcaaaaaatgaacaataactttcttgcacaactcggcggcttaac
ssTorA(Δ5-9) fw	ctagtctagataacgagggcaaaaaatgaacaataactcacgtcggcgttttctggc
ssTorA(Δ5-14) fw	ctagtctagataacgagggcaaaaaatgaacaataacctggcacaactcggcggc
ssTorA(Δ5-19) fw	ctagtctagataacgagggcaaaaaatgaacaataacggcttaaccgtggccggg

Table continues on next page

Primer name	Primer sequence (5' → 3')
ssTorA(Δ5-24) fw	ctagtctagataacgagggcaaaaaatgaacaataacgggatgctggggccgtca
ssTorA(Δ5-29) fw	ctagtctagataacgagggcaaaaaatgaacaataactcattgttaacgccgcgacg
ssTorA(Δ5-34) fw	ctagtctagataacgagggcaaaaaatgaacaataaccgacgtgctagcgcatctg
ssTorA(Δ5-39) fw	ctagtctagataacgagggcaaaaaatgaacaataactctgataaaattattcacctgac
ssTorA(Δ33-36) rv	gcatcggctagctaacaatgacggccccagcatc
ssTorA(Δ29-36) rv	gcatcggctagcccccagcatcccggccac
ssTorA(Δ25-36) rv	gcatcggctagcggccacggttaagccgcc
ssTorA(Δ21-36) rv	gcatcggctagcgccgccgagttgtgccaga
ssTorA(RR/AA) fw	ctctttcaggcatcagctgcgcgttttctggcacaac
ssTorA(RR/AA) rv	gttgtgccagaaaacgcgcagctgatgcctgaaagag
ssTorA(A16P) fw	gcgttttctgccacaactcgg
ssTorA(A16P) rv	ccgagttgtggcagaaaacgc
ssTorA(F14S) fw	gtcggcgttctctggcacaac
ssTorA(F14S) rv	gttgtgccagagaacgccgac
ssTorA(F7A-F14S) fw	caataacgatctcgctcaggcatcacgtcggcgttctctggcacaac
ssTorA(F7A-F14S) rv	gttgtgccagagaacgccgacgtgatgcctgagcgagatcgttattg
ssTorA(R11C) fw	caggcatcatgtcggcgttttc
ssTorA(R11C) rv	gaaaacgccgacatgatgcctg
ssTorA(R11H) fw	caggcatcacatcggcgttttc
ssTorA(R11H) rv	gaaaacgccgatgtgatgcctg
ssTorA(R11S) fw	caggcatcaagtcggcgttttc
ssTorA(R11S) rv	gaaaacgccgacttgatgcctg
ssTorA(R12L) fw	ggcatcacgtctgcgttttctgg
ssTorA(R12L) rv	ccagaaaacgcagacgtgatgcc
ssTorA(R12) fw	ggcatcacgtcagcgttttctgg
ssTorA(R12Q) rv	ccagaaaacgctgacgtgatgcc
ssTorA(L15Q) fw	gtcggcgttttcaggcacaactcg
ssTorA(L15Q) rv	cgagttgtgcctgaaaacgccgac
ssTorA(L15P) fw	gtcggcgttttccggcacaactcg
ssTorA(L15P) rv	cgagttgtgccggaaaacgccgac
ssTorA(L18H) fw	ctggcacaacacggcggcttaac
ssTorA(L18H) rv	gttaagccgccgtgttgtgccag
ssTorA(L18P) fw	ctggcacaacccggcggcttaac

Table continues on next page

**Primer name**	**Primer sequence (5' → 3')**
ssTorA(L18P) rv	gttaagccgccgggttgtgccag
ssTorA(G19D) fw	gcacaactcgacggcttaacc
ssTorA(G19D) rv	ggttaagccgtcgagttgtgc
ssTorA(L15M) fw	gtcggcgttttatggcacaac
ssTorA(L15M) rv	gttgtgccataaaacgccgac
ssTorA(Q17E) fw	gttttctggcagaactcggcggcttaac
ssTorA(Q17E) rv	gttaagccgccgagttctgccagaaaac
ssTorA(L18I) fw	ctggcacaaatcggcggcttaac
ssTorA(L18I) rv	gttaagccgccgatttgtgccag
ssTorA(L18V) fw	ctggcacaagtcggcggcttaac
ssTorA(L18V) rv	gttaagccgccgacttgtgccag
ssTorA(G19V) fw	gcacaactcgtcggcttaaccgtgg
ssTorA(G19V) rv	ccacggttaagccgacgagttgtgc
XbaI-RBS-CAT fw	gtcatctagataacgagggcaaaaaatggagaaaaaaatcactggatatac
*Hin*dIII-CAT rv	gactaagcttacgccccgccctgccac
*Nhe*I-CAT fw	gacgtgctagcgcaatggagaaaaaaatcactggatatac
*Spe*I_ssTorA rep fw	tgcatactagtatgaacaataacgatctctttc
*Nde*I MBP fw	aaggagatatacatatgaaaatcgaagaaggtaaactg
*Bam*HI MBP rv	cggtacccggggatccttacttggtgatacgagtctgc
ssTorA mut fw	ccactccctatcagtgatag
ssTorA mut rv	cgtgtcaaaactgtcgtcag
ssTorA MiSeq fw	cctatcagtgatagagaaaag
ssTorA MiSeq rv	gatatatcaacggtggtatatc
qPCR TrxA fw	gtcgatttctgggcagagtg
qPCR TrxA rv	gcagcagagtcgggatacc
qPCR Bla fw	catcttacggatggcatgac
qPCR Bla rv	cgatcaaggcgagttacatg

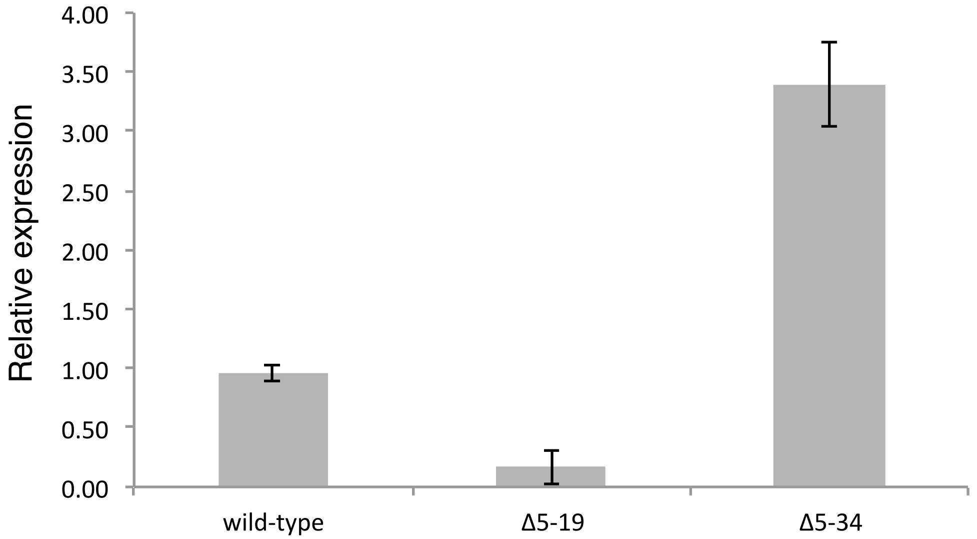

Supplementary Figure 1. mRNA levels for ssTorA/TrxA deletion mutants. Quantitive PCR analysis of *trxA* mRNA expression levels driven by pIBA-ssTorA/TrxA, pIBA-ssTorA(Δ5-19) and pIBA-ssTorA(Δ5-34). Expression levels relative to those of *bla* present as an antibiotic resistance marker on the respective pIBA vector backbones were determined. Mean plus or minus SD values of PCR duplicates are shown.

A

```
TRANSLATION      M  N  N  N  D  L  F  Q  A  S  R  R  R  F  L  A  Q  L  G  G  L  T  V  A  G  M  L  G  P  S  L  L  T  P
3X               ATGAACAATAACGATCTCTTTCAGGCATCACGTCGGCGTTTTCTGGCACAACTCGGCGGCTTAACCGTGGCCGGGATGCTGGGGCCGTCATTGTTAACGC
Δ29/36|3X;NO     ATGAACAATAACGATCTCTTTCAGGCATCACGTCGGCGTTTTCTGGCACAACTCGGCGGCTTAACCGTGGCCGGGATGCTGGGG----------------
Δ29/36|3X        ATGAACAATAACGATCTGTTTCAAGCAAGTCGCCGCCGCTTCCTGGCGCAGTTAGGCGGCTTACAGTCGCGGGTATGTTGGGA----------------
                 **************** ***** ***   ** ** ** ** ***** **  * *****  * ** ** ** ** *** ****

TRANSLATION        R  R  A  S  M  N  N  N  D  L  F  Q  A  S  R  R  R  F  L  A  Q  L  G  G  L  T  V  A  G  M  L  G  P
3X               CGCGACGTGCTAGTATGAACAATAACGATCTCTTTCAGGCATCACGTCGGCGTTTTCTGGCACAACTCGGCGGCTTAACCGTGGCCGGGATGCTGGGGCC
Δ29/36|3X;NO     --------GCTAGTATGAACAATAACGATCTCTTTCAGGCATCACGTCGGCGTTTTCTGGCACAACTCGGCGGCTTAACCGTGGCCGGGATGCTGGGG--
Δ29/36|3X        --------------ATGAACAATAACGATCTTTTTCAGGCTTCCCGCCGCCGTTTTTTGGCCCAATTAGGCGGCTTAACTGTAGCCGGGATGTTAGGT--
                               **************** ******** ** ** ** ****** **** *** * *********** ** ********* * **

TRANSLATION       S  L  L  T  P  R  R  A  S  M  N  N  N  D  L  F  Q  A  S  R  R  R  F  L  A  Q  L  G  G  L  T  V  A
3X               GTCATTGTTAACGCCGCGACGTGCTAGTATGAACAATAACGATCTCTTTCAGGCATCACGTCGGCGTTTTCTGGCACAACTCGGCGGCTTAACCGTGGCC
Δ29/36|3X;NO     ----------------------GCTAGTATGAACAATAACGATCTCTTTCAGGCATCACGTCGGCGTTTTCTGGCACAACTCGGCGGCTTAACCGTGGCC
Δ29/36|3X        ----------------------------ATGAATAATAACGACTTATTCCAGGCGAGCCGTCGCCGCTTCTTAGCGCAGTTGGGGGGGTTAACCGTTGCT
                                             ***** ********   * **   *****   ***** ** **   * ** **   * ** ** ******** **

TRANSLATION      G  M  L  G  P  S  L  L  T  P  R  R  A  S  A
3X               GGGATGCTGGGGCCGTCATTGTTAACGCCGCGACGTGCTAGCGCA
Δ29/36|3X;NO     GGGATGCTGGGG------------------------GCTAGCGCA
Δ29/36|3X        GGAATGCTGGGC------------------------GCTAGCGCA
                 ** ********                         *********
```

B

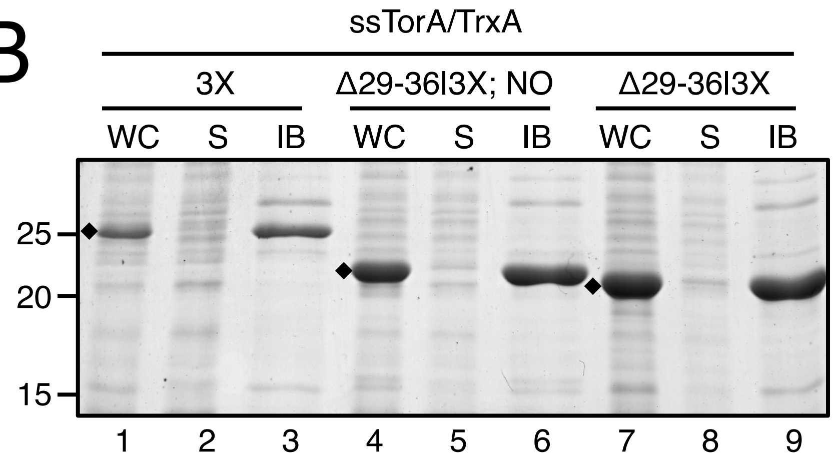

Supplementary Figure 2. Influence of codon optimization on expression and IB formation. (A) Alignment of the DNA coding sequence for ssTorA(3X) versus *E. coli* optimized and non-optimized (NO) coding sequences for truncated derivative ssTorA(Δ29-36|3X). **(B)** IB-sedimentation assay as described in the legend to Figure 1 on cells expressing TrxA fused to ssTorA(3X) or ssTorA(Δ29-36|3X) encoded by the respective DNA sequences under A. Bands representing fusion proteins of interest are indicated (*diamond*). Molecular mass (kDa) markers are indicated at the left side of the panels.

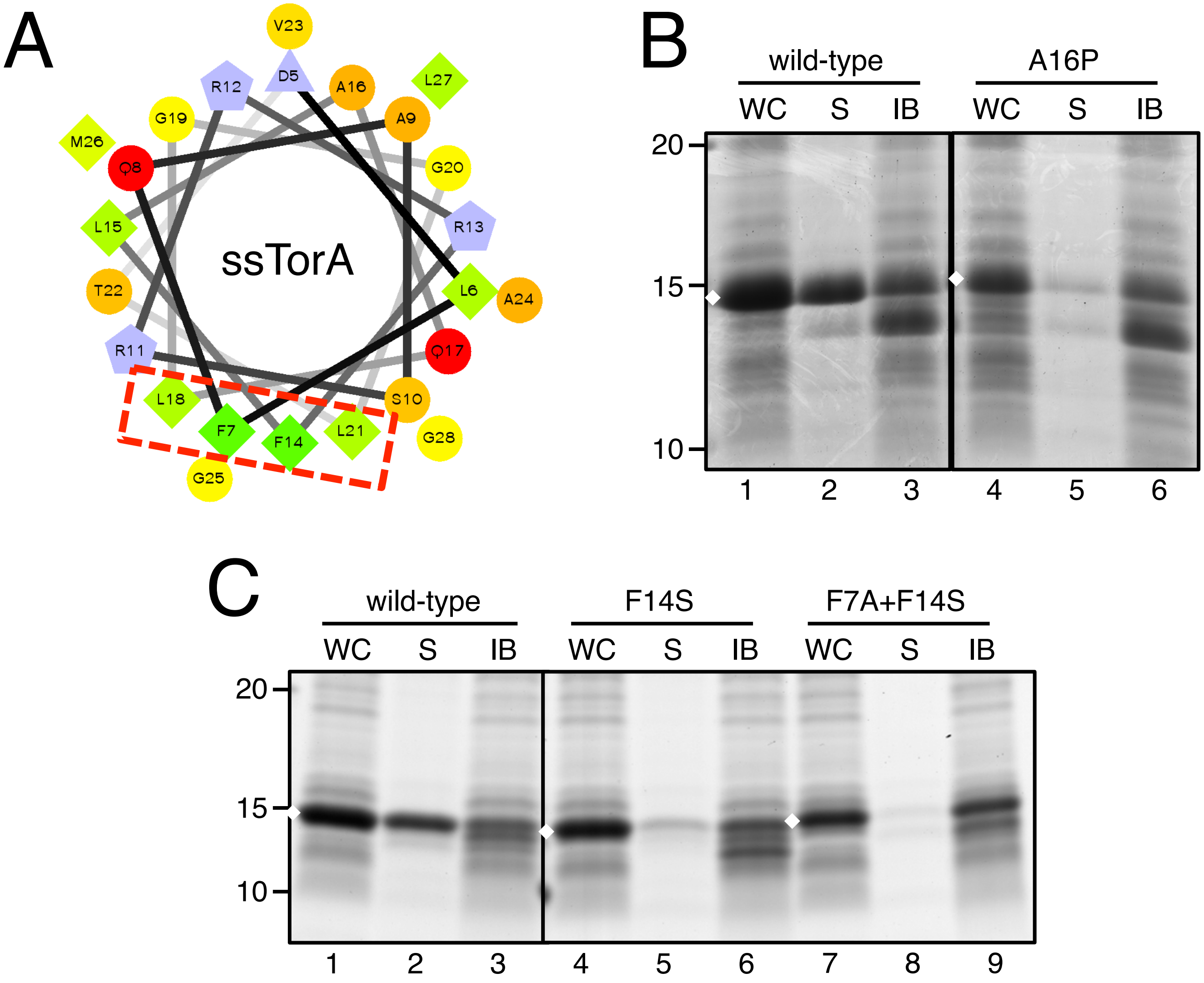

Supplementary Figure 3. Influence of α-helix formation on IB formation. (A) Helical wheel representation of ssTorA (AA 5-28) (http://rzlab.ucr.edu/scripts/wheel/wheel.cgi). A concentration of hydrophobic residues (F7-F14-L18-L21) that may form upon assembly of an α-helix is enclosed in a red dashed box. **(B and C)** IB-sedimentation assay as described in the legend to Fig. 1 on cells expressing (B) ssTorA/TrxA (wild-type) or ssTorA(A16P)/TrxA and (C) ssTorA/TrxA (wild-type), ssTorA(F14S)/TrxA or ssTorA(F7A+F14S)/TrxA. Bands representing fusion proteins of interest are indicated (*diamond*). Molecular mass (kDa) markers are indicated at the left side of the panels.

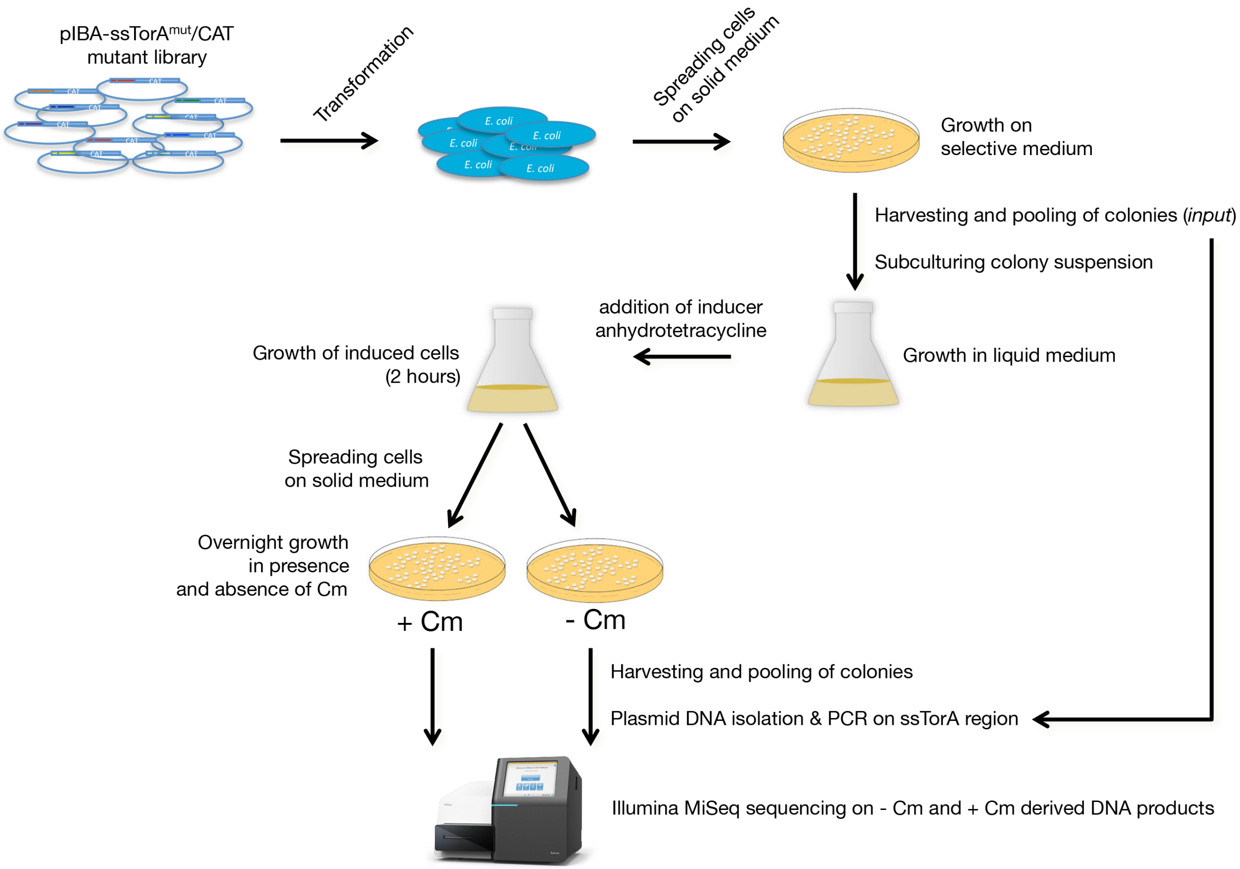

Supplementary Figure 4. Set-up of (in)solubility screen. A pIBA-ssTorA/CAT-based library carrying a randomly mutagenized ssTorA^{mut}-coding sequence is transformed into *E. coli* TOP10F'. Following growth of the transformed cells on plates with ampicillin to select for the presence of pIBA-ssTorA/CAT, emerging colonies are scrape-harvested and pooled to a suspension (*input*). To start (in)solubility screening, part of the suspension is used to inoculate a culture in LB-medium containing ampicillin. When the exponential growth phase is reached ($OD^{660} = 0.3$) cells are induced for overexpression of ssTorA^{mut}/CAT by addition of anhydrotetracyclin. After 2 hours part of the culture is spread on LB-agar/ampicillin plates containing chloramphenicol (*Cm*) to screen for the presence of cells expressing ssTorA^{mut}/CAT in a soluble fashion by virtue of the chloramphenicol-inactivating capacity of CAT when soluble. In parallel, another part of the culture is spread on LB-agar/ampicillin plates without chloramphenicol to sustain growth of both cells expressing ssTorA^{mut}/CAT in soluble or insoluble fashion, respectively. Colonies emerging following overnight growth in the presence or absence of chloramphenicol are scrape-harvested and pooled to separate suspensions (+ Cm and – Cm). Both suspensions, as well as the input suspension, are subjected to plasmid extraction and PCR is carried out to amplify the ssTorA^{mut}-coding sequence of the respective plasmid isolates. PCR products are subjected to Illumina MiSeq sequencing to determine the mutational loads in the ssTorA segments of the respective cell pools.

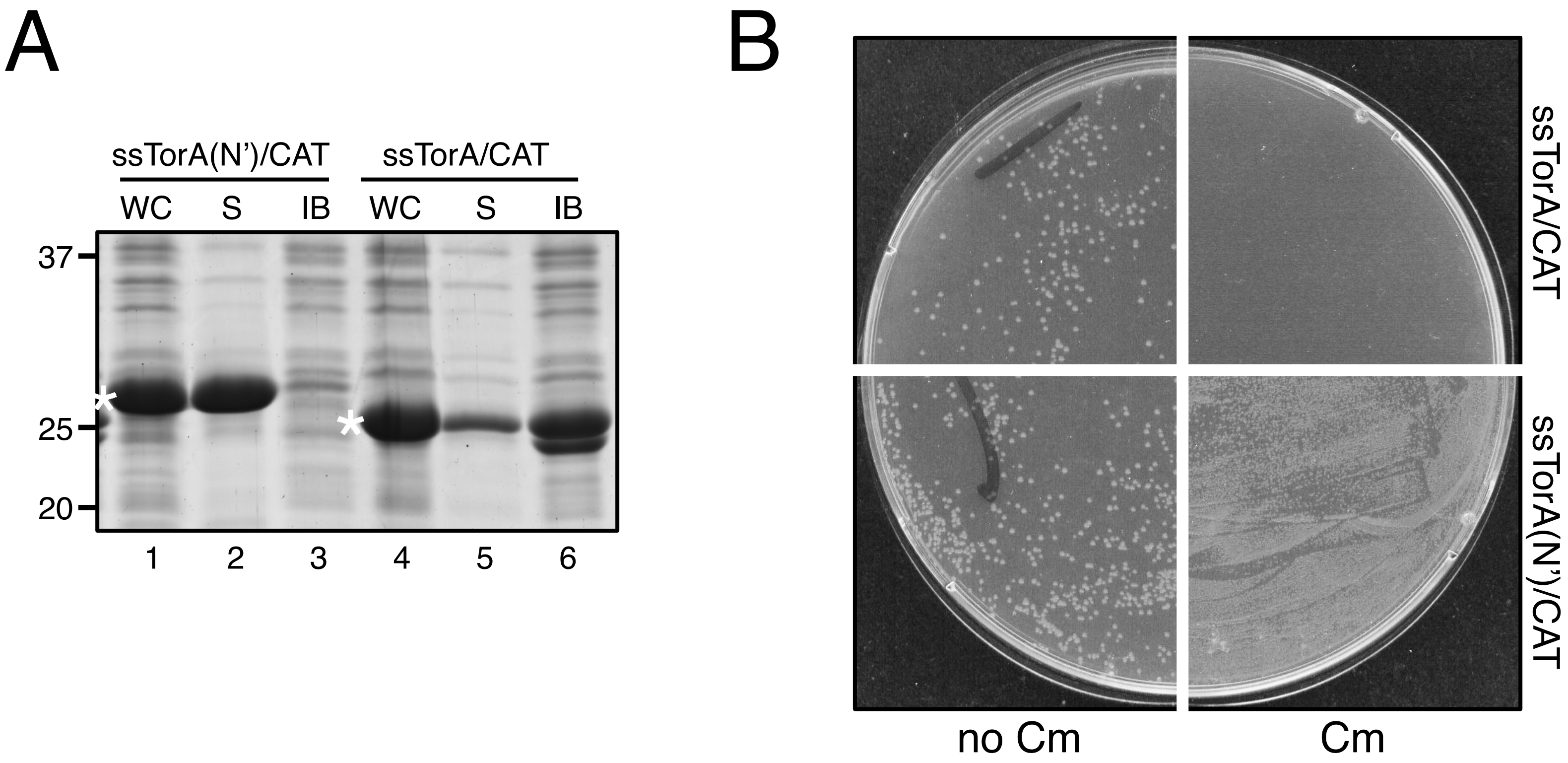

Supplementary Figure 5. Influence of ssTorA on solubility of CAT. (A) IB-sedimentation assay as described in the legend to Figure 1 on cells expressing ssTorA(N')/CAT or ssTorA/CAT. Bands representing fusion proteins of interest are indicated (*). Molecular mass (kDa) markers are indicated at the left side of the panel. **(B)** Colony formation upon overnight growth of cells expressing either ssTorA(N')/CAT or ssTorA/CAT on LB-agar medium in the presence or absence of 30 µg/ml chloramphenicol (Cm). Cells were first grown in liquid medium and induced for protein overexpression with anhydrotetracycline for 2 hours before growth was continued on solid medium. Approximately 10^6 and 10^4 cells were spread on plates with and without Cm, respectively.

Ref pos	Ref seq	Translation	Mutation frequency			Features
			Input	*- Cm*	*+ Cm*	
1	T		0.00	0.00	0.00	XbaI
2	C		0.00	0.00	0.00	
3	T		0.00	0.00	0.00	
4	A		0.00	0.00	0.00	
5	G		0.00	0.00	0.00	
6	A		0.01	0.01	0.00	
7	T		0.02	0.02	0.02	
8	A		0.01	0.01	0.01	
9	A		0.02	0.02	0.00	Shine-Dalgarno
10	C		0.04	0.04	0.02	
11	G		0.02	0.03	0.03	
12	A		0.02	0.02	0.00	
13	G		0.01	0.01	0.00	
14	G		0.03	0.04	0.01	
15	G		0.04	0.04	0.05	
16	C		0.03	0.03	0.06	
17	A		0.01	0.01	0.01	
18	A		0.02	0.01	0.02	
19	A		0.01	0.00	0.01	
20	A		0.01	0.02	0.00	
21	A		0.07	0.08	0.04	
22	A	Met - 1	0.08	0.08	0.07	
23	T		0.02	0.02	0.01	
24	G		0.02	0.03	0.01	
25	A	Asn - 2	0.01	0.01	0.01	
26	A		0.02	0.02	0.02	
27	C		0.03	0.02	0.04	
28	A	Asn -3	0.02	0.02	0.01	
29	A		0.02	0.02	0.02	
30	T		0.02	0.02	0.01	
31	A	Asn - 4	0.01	0.01	0.00	
32	A		0.02	0.03	0.05	
33	C		0.03	0.03	0.04	
34	G	Asp - 5	0.03	0.03	0.03	
35	A		0.02	0.01	0.01	
36	T		0.02	0.02	0.02	
37	C	Leu - 6	0.03	0.04	0.01	
38	T		0.03	0.02	0.03	
39	C		0.02	0.01	0.04	
40	T	Phe - 7	0.02	0.02	0.02	
41	T		0.02	0.02	0.03	
42	T		0.02	0.02	0.04	
43	C	Gln - 8	0.01	0.01	0.04	
44	A		0.02	0.02	0.02	
45	G		0.04	0.04	0.02	
46	G	Ala - 9	0.04	0.04	0.05	
47	C		0.03	0.02	0.03	
48	A		0.03	0.03	0.03	

Ref pos	Ref seq	Translation	Mutation frequency			Features
			Input	*- Cm*	*+ Cm*	
49	T	Ser - 10	0.02	0.02	0.03	
50	C		0.02	0.02	0.04	
51	A		0.02	0.02	0.02	
52	C	Arg - 11	0.03	0.03	0.08	
53	G		0.04	0.04	0.17	
54	T		0.02	0.02	0.01	
55	C	Arg - 12	0.03	0.03	0.04	
56	G		0.05	0.05	0.13	
57	G		0.03	0.02	0.01	
58	C	Arg - 13	0.04	0.04	0.02	
59	G		0.05	0.05	0.06	
60	T		0.03	0.03	0.04	
61	T	Phe - 14	0.02	0.02	0.04	
62	T		0.06	0.07	0.08	
63	T		0.02	0.02	0.03	
64	C	Leu - 15	0.03	0.03	0.02	
65	T		0.01	0.01	0.12	
66	G		0.02	0.02	0.03	
67	G	Ala - 16	0.05	0.05	0.04	
68	C		0.03	0.03	0.02	
69	A		0.02	0.02	0.00	
70	C	Gln - 17	0.03	0.04	0.00	
71	A		0.01	0.01	0.01	
72	A		0.02	0.02	0.01	
73	C	Leu - 18	0.04	0.04	0.02	
74	T		0.04	0.04	0.19	
75	C		0.04	0.04	0.04	
76	G	Gly - 19	0.05	0.05	0.06	
77	G		0.03	0.03	0.12	
78	C		0.03	0.03	0.01	
79	G	Gly - 20	0.06	0.06	0.05	
80	G		0.05	0.05	0.10	
81	C		0.04	0.04	0.04	
82	T	Leu - 21	0.02	0.02	0.05	
83	T		0.01	0.01	0.01	
84	A		0.01	0.01	0.01	
85	A	Thr - 22	0.02	0.02	0.02	
86	C		0.02	0.02	0.03	
87	C		0.04	0.04	0.02	
88	G	Val - 23	0.04	0.04	0.04	
89	T		0.04	0.04	0.06	
90	G		0.02	0.02	0.01	
91	G	Ala - 24	0.04	0.04	0.04	
92	C		0.03	0.03	0.02	
93	C		0.02	0.02	0.04	
94	G	Gly - 25	0.03	0.03	0.03	
95	G		0.04	0.04	0.04	
96	G		0.03	0.02	0.03	

Ref pos	Ref seq	Translation	Mutation frequency			Features
			Input	*- Cm*	*+ Cm*	
97	A	Met - 26	0.02	0.02	0.02	
98	T		0.02	0.02	0.03	
99	G		0.04	0.04	0.04	
100	C	Leu - 27	0.05	0.05	0.07	
101	T		0.02	0.02	0.06	
102	G		0.01	0.00	0.01	
103	G	Gly - 28	0.03	0.04	0.04	
104	G		0.07	0.07	0.05	
105	G		0.04	0.05	0.04	
106	C	Pro - 29	0.03	0.03	0.02	
107	C		0.03	0.03	0.02	
108	G		0.04	0.04	0.04	
109	T	Ser - 30	0.02	0.02	0.04	
110	C		0.01	0.01	0.03	
111	A		0.03	0.03	0.03	
112	T	Leu - 31	0.02	0.02	0.04	
113	T		0.02	0.02	0.01	
114	G		0.03	0.03	0.04	
115	T	Leu - 32	0.02	0.02	0.01	
116	T		0.00	0.00	0.01	
117	A		0.01	0.01	0.01	
118	A	Thr - 33	0.02	0.02	0.01	
119	C		0.04	0.04	0.03	
120	G		0.04	0.04	0.05	
121	C	Pro - 34	0.01	0.03	0.01	
122	C		0.03	0.03	0.04	
123	G		0.05	0.04	0.05	
124	C	Arg - 35	0.03	0.03	0.01	
125	G		0.03	0.03	0.02	
126	A		0.02	0.02	0.02	
127	C	Arg - 36	0.04	0.04	0.04	
128	G		0.06	0.06	0.07	
129	T		0.07	0.08	0.01	
130	G	Ala - 37	0.00	0.00	0.01	NheI
131	C		0.00	0.00	0.00	
132	T		0.00	0.00	0.01	
133	A	Ser - 38	0.00	0.00	0.00	
134	G		0.00	0.00	0.01	
135	C		0.00	0.00	0.00	
136	G	Ala - 39	0.01	0.01	0.00	
137	C		0.00	0.00	0.00	
138	A		0.00	0.00	0.00	

Supplementary Figure 6. Mutational loads in Cm-selected versus non-selected cells. Mutation frequencies per position of the reference sequence (non ref counts/[ref counts + non ref counts]) as determined by Illumina MiSeq-sequencing. Below average frequencies are shaded in green, above average frequencies are shaded in red. Positions showing frequencies above 0.1 in the Cm-selected sample (+ *Cm*) are deemed mutation hotspots. Corresponding amino acids are colored red in the translation of the ssTorA coding sequence.

Ref pos	Ref seq	Translation	*Mutation frequency - Cm*				*Mutation frequency + Cm*				Amino Acid Substitut
			A	C	G	T	A	C	G	T	
52	C	Arg - 11	0.008	0.970	0.002	0.020	0.040	0.920	0.002	0.037	Ser, Cys
53	G		0.025	0.006	0.961	0.008	0.136	0.007	0.834	0.024	His
54	T		0.007	0.004	0.004	0.984	0.008	0.002	0.002	0.987	
55	C	Arg - 12	0.012	0.973	0.004	0.011	0.013	0.957	0.007	0.022	
56	G		0.019	0.005	0.953	0.019	0.078	0.014	0.870	0.038	Gln, Leu
57	G		0.014	0.002	0.982	0.002	0.010	0.001	0.988	0.001	
58	C	Arg - 13	0.011	0.963	0.007	0.019	0.001	0.978	0.015	0.006	
59	G		0.019	0.006	0.951	0.024	0.005	0.031	0.938	0.026	
60	T		0.007	0.008	0.011	0.975	0.014	0.012	0.012	0.962	
61	T	Phe - 14	0.009	0.003	0.008	0.979	0.019	0.008	0.004	0.962	
62	T		0.014	0.007	0.005	0.935	0.019	0.040	0.016	0.925	
63	T		0.004	0.004	0.008	0.978	0.025	0.001	0.002	0.970	
64	C	Leu - 15	0.012	0.973	0.002	0.012	0.012	0.976	0.007	0.005	Met
65	T		0.005	0.004	0.004	0.987	0.060	0.042	0.018	0.880	Gln, Pro
66	G		0.008	0.004	0.976	0.011	0.011	0.008	0.975	0.006	
67	G	Ala - 16	0.032	0.003	0.954	0.010	0.026	0.005	0.959	0.010	
68	C		0.005	0.967	0.007	0.021	0.002	0.977	0.013	0.008	
69	A		0.980	0.010	0.003	0.006	0.996	0.003	0.000	0.000	
70	C	Gln - 17	0.009	0.959	0.002	0.030	0.002	0.997	0.000	0.000	
71	A		0.988	0.004	0.004	0.004	0.989	0.005	0.001	0.005	
72	A		0.975	0.005	0.008	0.010	0.991	0.001	0.002	0.005	
73	C	Leu - 18	0.019	0.961	0.003	0.016	0.007	0.980	0.001	0.012	Ile, Val
74	T		0.022	0.014	0.002	0.962	0.080	0.086	0.023	0.811	His, Pro
75	C		0.023	0.961	0.004	0.012	0.009	0.963	0.008	0.020	
76	G	Gly - 19	0.022	0.007	0.954	0.015	0.022	0.015	0.942	0.021	
77	G		0.023	0.006	0.967	0.002	0.108	0.008	0.884	0.000	Asp, Val
78	C		0.005	0.968	0.005	0.021	0.002	0.988	0.000	0.009	

Supplementary Figure 7. Determination of favored and non-favored substitutions for interference with IB tag functionality. Frequency of occurrence of individual nucleotides (A, C, G, T) per position of the reference sequence for ssTorA AA 11 – 19 (nucleotide count/[non ref count + ref count]) as determined by Illumina MiSeq-sequencing. Shaded in red are nucleotide substitutions occuring with elevated frequency in the Cm-selected sample (+ Cm) compared to the non-selected sample (- Cm) and giving rise to amino acid substitutions potentially interfering with the functionality of the ssTorA IB formation tag. Corresponding amino acid substitutions are displayed in red. Shaded in green are selected nucleotide substitutions in the + Cm sample occurring with unchanged or lowered frequency compared to the – Cm sample and giving rise to amino acid substitutions that should not interfere with the functionality of the ssTorA tag. Corresponding amino acid substitutions are displayed in green.

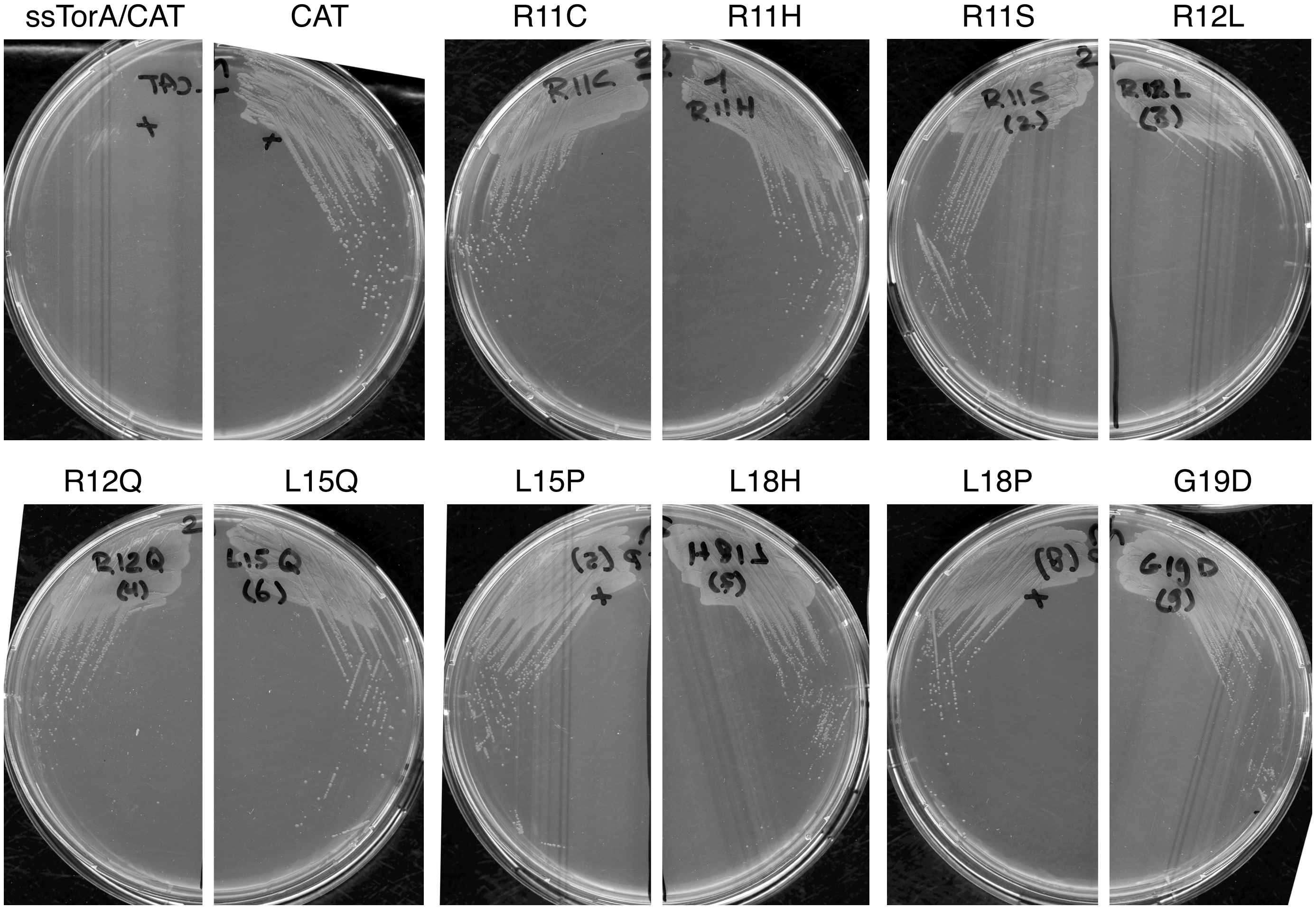

Supplementary Figure 8. On-plate chloramphenicol resistance upon expression of ssTorA/CAT mutants. Colony formation on LB-agar containing chloramphenicol upon growth of cells expressing ssTorA/CAT derivatives carrying the indicated single amino acid substitutions in the ssTorA moiety. Cells were first grown in liquid medium and induced for protein overexpression with anhydrotetracycline for 2 hours before being streaked on solid medium for overnight growth at 37°C. As controls, expression of ssTorA/CAT carrying wild-type ssTorA does not support colony formation in the presence of chloramphenicol, whereas expression of CAT lacking ssTorA (CAT) does.

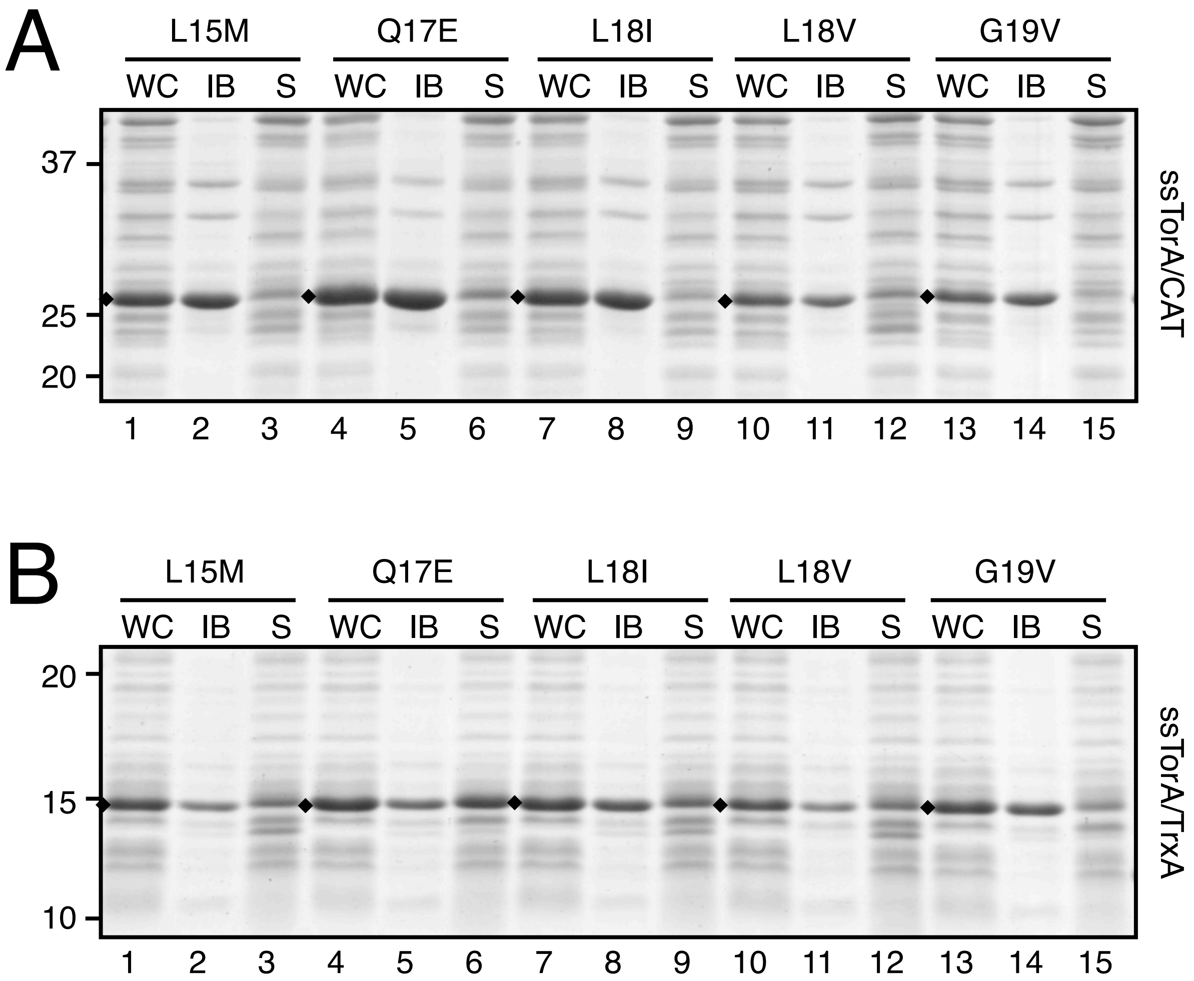

Supplementary Figure 9. Influence of potential non-solubilizing substitutions on IB formation. IB-sedimentation assay as described in the legend to Fig. 1 on cells expressing ssTorA/CAT **(A)** or ssTorA/TrxA **(B)** derivatives carrying the indicated single amino acid substitutions in the ssTorA moiety. Bands representing fusion proteins of interest are indicated (*diamond*). Molecular mass (kDa) markers are indicated at the left side of the panels.

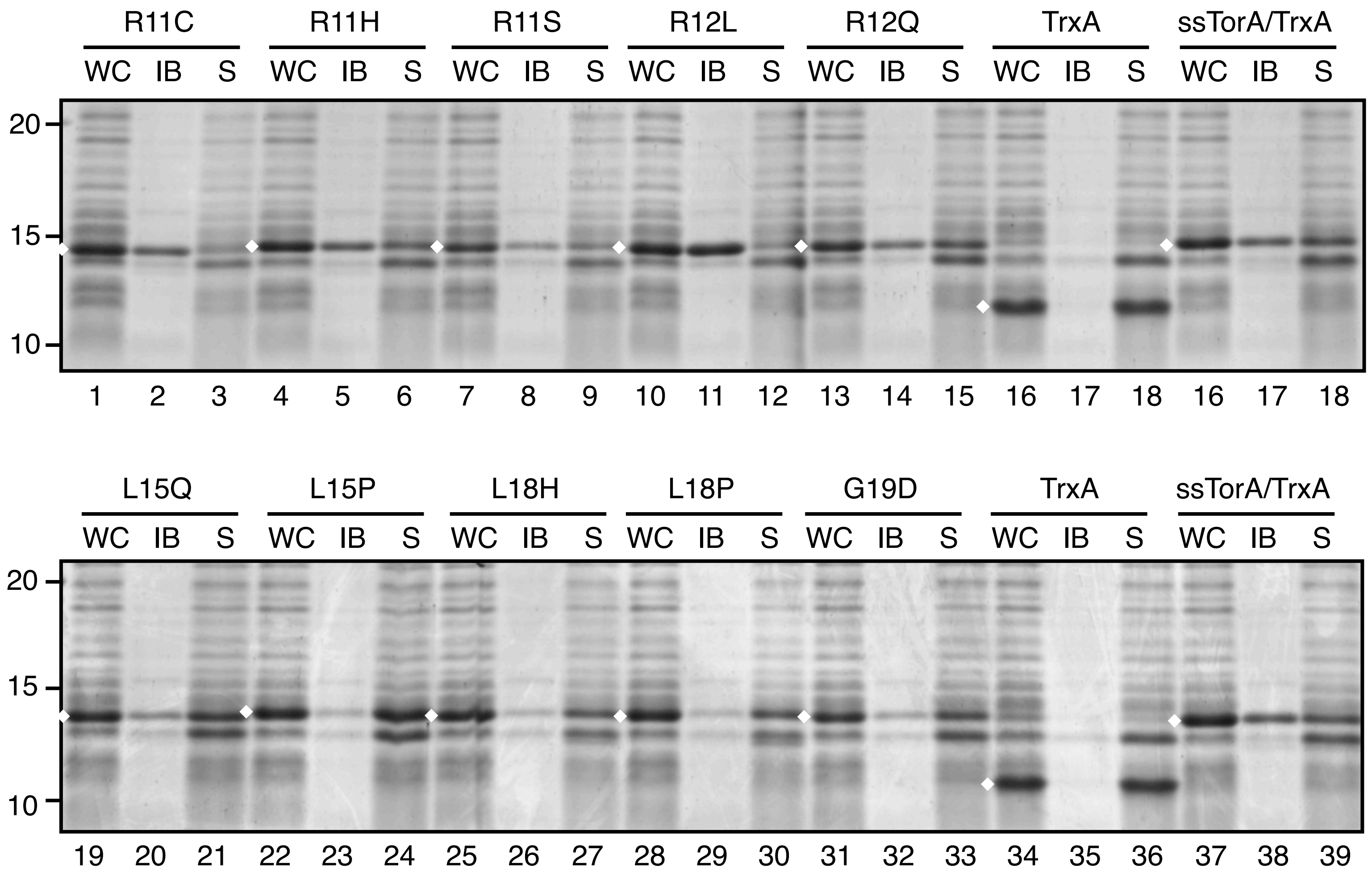

Supplementary Figure 10. Influence of potential solubilizing substitutions on IB formation of ssTorA/TrxA. IB-sedimentation assay as described in the legend to Figure 1 on cells expressing ssTorA/TrxA derivatives carrying the indicated single amino acid substitutions in the ssTorA moiety. ssTorA/TrxA carrying wild-type ssTorA and TrxA lacking ssTorA (TrxA) were analyzed in parallel as controls. Bands representing fusion proteins of interest are indicated (*diamond*). Molecular mass (kDa) markers are indicated at the left side of the panels.
